# Distribution of Genetic Determinants Associated with CRISPR-Cas Systems and Resistance to Antibiotics in the Genomes of Archaea and Bacteria

**DOI:** 10.3390/microorganisms13061321

**Published:** 2025-06-06

**Authors:** Laura Antequera-Zambrano, Ángel Parra-Sánchez, Lenin González-Paz, Eduardo Fernandez, Gema Martinez-Navarrete

**Affiliations:** 1 Genetics and Molecular Biology Laboratory, Biology Department, Universidad of Zulia, Maracaibo 4001, Venezuela; 2 Bioengineering Institute, University Miguel Hernández of Elche, 03202 Elche, Spain; a.parra@umh.es (Á.P.-S.); e.fernandez@umh.es (E.F.); 3 Laboratory of Modeling, Dynamics, and Subcellular Biochemistry (LMDBS), Center for Molecular Biomedicine (CBM), Venezuelan Institute for Scientific Research (IVIC), Maracaibo 4001, Venezuela; lgonzalezpaz@gmail.com; 4 Biomedical Research Network Center (CIBER-BBN), 28029 Madrid, Spain; 5 Institute for Health and Biomedical Research (ISABIAL), Dr. Balmis General and University Hospital, 03000 Alicante, Spain

**Keywords:** CRISPR-Cas, resistance to antibiotics, Archaea and Bacteria, horizontal gene transfer

## Abstract

The CRISPR-Cas system represents an adaptive immune mechanism found across diverse Archaea and Bacteria, allowing them to defend against invading genetic elements such as viruses and plasmids. Despite its broad distribution, the prevalence and complexity of CRISPR-Cas systems differ significantly between these domains. This study aimed to characterize and compare the genomic distribution, structural features, and functional implications of CRISPR-Cas systems and associated antibiotic resistance genes in 30 archaeal and 30 bacterial genomes. Through bioinformatic analyses of CRISPR arrays, *cas* gene architectures, direct repeats (DRs), and thermodynamic properties, we observed that Archaea exhibit a higher number and greater complexity of CRISPR loci, with more diverse cas gene subtypes exclusively of Class 1. Bacteria, in contrast, showed fewer CRISPR loci, comprising a mix of Class 1 and Class 2 systems, with Class 1 representing the majority (~75%) of the detected systems. Notably, Bacteria lacking CRISPR-Cas systems displayed a higher prevalence of antibiotic resistance genes, suggesting a possible inverse correlation between the presence of these immune systems and the acquisition of such genes. Phylogenetic and thermodynamic analyses further highlighted domain-specific adaptations and conservation patterns. These findings support the hypothesis that CRISPR-Cas systems play a dual role: first, as a defense mechanism preventing the integration of foreign genetic material—reflected in the higher complexity and diversity of CRISPR loci in Archaea—and second, as a regulator of horizontal gene transfer, evidenced by the lower frequency of antibiotic resistance genes in organisms with active CRISPR-Cas systems. Together, these results underscore the evolutionary and functional diversification of CRISPR-Cas systems in response to environmental and selective pressures.

## 1. Introduction

The Archaea and Bacteria domains are estimated to have emerged around 3.6 to 4 billion years ago and can live in extreme habitats [1,2]. There is limited genetic information on Archaea due to the difficulty of characterizing these organisms, which is hindered by the inability to cultivate them in the laboratory by conventional methods [3]. Recent studies using uniquely conserved gene copies suggest that Archaea are the closest relatives of eukaryotes, which is why phylogenetic studies are still ongoing [4,5]. Bacteria, on the other hand, are often present and utilized in various biotechnologically relevant environments. Over time, Bacteria have developed diverse molecular resistance mechanisms that impact their control and biotechnological applications, while also contributing to the emergence of severe infectious diseases [6]. In contrast, Archaea play essential roles in anaerobic wastewater purification and the global carbon and nitrogen cycles, processes that are highly relevant to agriculture and environmental sustainability. Bacteria, meanwhile, have a dual significance: they are key agents in industrial and food biotechnology—used in the production of antibiotics, insecticides, fermented foods like yogurt, and the conversion of ethanol into acetic acid [7,8,9,10] —but they are also major pathogens with increasing antimicrobial resistance [11,12]. The spread of antibiotic resistance genes among Bacteria is a critical global health concern, especially in developing countries where limited access to effective treatment accelerates dissemination [11,13,14]. This resistance, shaped by evolutionary pressure from antibiotics, antiseptics, and disinfectants, affects both human pathogens and environmental microorganisms [13,15].

Additionally, Archaea and Bacteria have developed an “adaptive immune system” consisting of clustered regularly interspaced short palindromic repeats (CRISPR) [16] to defend themselves against foreign genetic material. This immune system is mediated by nucleases (CAS) that degrade invading genetic material, and some fragments of the degraded molecule are subsequently stored to recognize and eliminate similar sequences in the future [17]. Despite extensive studies on this technology, many questions about the biology of CRISPR-Cas and its complete functionality remain unanswered [18,19,20,21]. It has been found that 90% of Archaea, especially hyperthermophiles, possess the CRISPR-Cas system, whereas only around 40% of Bacteria have it, along with other defense systems, such as restriction–modification, which is nearly ubiquitous among prokaryotes except for certain parasitic Bacteria [22]. There is limited evidence explaining why Archaea preferentially retain CRISPR-Cas systems, while Bacteria often rely on alternative defense mechanisms [20,23,24,25]. However, it is thought that Archaea are more frequently exposed to viral attacks than Bacteria because, over time, Bacteria have diversified more extensively in their habitats and encountered different threats like antibiotics [26]. This biological system is an important one to study for allowing for the storage of information from past viral infections to recognize and neutralize invading sequences in a specific manner [27]. Archaea and Bacteria are not only threatened by their viruses (known as bacteriophages) but are also exposed to the incorporation of other genetic material like plasmids. These plasmids can be beneficial or harmful depending on the genetic information they carry during processes known as horizontal gene transfer. This averages that DNA is transferred between different cells rather than being vertically passed to offspring, which significantly contributes to bacterial evolution and is a critical factor in the widespread dissemination of antibiotic resistance genes [28,29,30]. There is evidence that the CRISPR-Cas system interferes with the horizontal transfer of antibiotic resistance genes, which demonstrates that CRISPR-Cas is not limited to phage defense [28,31,32]. Although many horizontally transferred genes are beneficial for Bacteria, others are not, and it is believed that the CRISPR-Cas system provides an average to limit or control the non-essential genetic information that microorganisms could accumulate.

Currently, various research studies and discoveries are being conducted on the CRISPR-Cas system and range from the comparative analysis of the CRISPR-Cas system in a bacterial genus [33] to entire groups of Archaea and Bacteria [34]. Research into its utility in genetic editing has also increased. However, more comparative studies related to the conservation of these structures between Archaea and Bacteria are needed to explain existing uncertainties about their prevalence [20]. Based on this, the objective of this study is to determine the distribution, diversity, and structural characteristics of CRISPR-Cas systems in Archaea and Bacteria, and to explore the possibility of interaction between these systems. We hypothesize that Archaea maintain more complex and diverse CRISPR-Cas architectures due to their adaptation to extreme environments and greater exposure to viral threats, whereas Bacteria exhibit higher variability in system presence driven by ecological pressures and the adoption of alternative defense mechanisms. Additionally, we propose that the presence or absence of CRISPR-Cas systems may influence the acquisition of horizontally transferred genes, including antibiotic resistance genes, particularly in Bacteria.

## 2. Materials and Methods

### 2.1. Obtaining Genomic Sequences from the Representative Genera of Archaea and Bacteria

First, 308 complete archaeal genomic sequences were analyzed and selected based on the following inclusion criteria: (1) fully assembled genomes (100%), (2) one representative genome per species to avoid redundancy, and (3) confirmed presence of CRISPR-Cas structures. To obtain bacterial genomes, the same number of archaeal genomes with confirmed CRISPR structures (30) was selected. This yielded a total of 60 complete genomes for comparative analysis (30 from Archaea and 30 from Bacteria). In addition, 30 bacterial genomes without confirmed CRISPR structures were selected using the same criteria—except for the absence of CRISPR systems—with an additional criterion: from different isolates. This allowed for comparison with environmental Bacteria to assess the variability of resistance genes in the presence or absence of CRISPR structures. These genomes were retrieved from the National Center for Biotechnology Information (NCBI, MD, USA) database (http://www.ncbi.nlm.nih.gov/ accessed on 25 July 2023) [35]. Genomic sequences were also obtained from specific sites listed in the Entrez Genome Project (http://www.ncbi.nlm.nih.gov/genomes/lproks.cgi accessed on 25 July 2023) and the Genomes OnLine Database (Joint Genome Institute, CA, USA) (http://www.genomesonline.org/, accessed on 30 August 2023) [36].

### 2.2. Identification of CRISPR Structures

In order to obtain DRs, CRISPR loci in genomes were identified using the CRISPRFinder v1.1.2 database (Institut de Biologie Intégrative de la Cellule, Université Paris-Saclay, Gif-sur-Yvette, France), accessed on 15 December 2023 [37]. Two search criteria were followed: screening for potential CRISPR locations through detection of maximal repeats (maximum extension repeat to the right or left without incurring a mismatch) using the VMatch 2.3.0 package, which is an updated version of REPuter [38] based on the efficient implementation of enhanced suffix arrays [39]. The employed default parameters were as follows: repeat length from 23 to 55 bp, gap size between repeats from 25 to 60 bp, and 20% nucleotide mismatch between repeats [37]. The other search criterion was based on determining CRISPR functions, for which filters were added to help validate a CRISPR, such as searching for unidentical spacers with a size that should go from 0.6 * to 2.5 * the repeat size. This filter is configured to eliminate tandem repeats.

To discriminate between the confirmed CRISPR structures and questionable ones, small structures resembling CRISPR, i.e., those with only two or three DRs, were classified using a level of evidence rated from 1 to 4. Level 1 includes small CRISPRs (with 3 or fewer spacers), while levels 2 to 4 are classified based on repeat and spacer similarity. Additionally, tests were considered to verify the internal conservation of the candidate repeats and spacer divergence provided by CRISPRFinder. This allowed for a more accurate identification of true CRISPR arrays.

### 2.3. Identification and Comparison of the Genes Associated with CRISPR and CAS Proteins

To search for *cas* genes, the first step involved identifying open reading frames (ORFs) using Prodigal v2.6.3 [40]. These ORFs were then analyzed by the MacSyFinder program using Hidden Markov Models (HMMs) for gene search in a library of known CAS proteins [41]. Alternatively, BLAST v2.15.0 (Basic Local Alignment Search Tool) was applied to identify *cas* genes in the sequences upstream and downstream of CRISPR loci, and TIGRFAM [42] was employed. CAS type and subtype were determined by a clustering analysis using the CRISPRCas-Finder v1.1.2 program [37].

Phylogenetic trees were generated based on the Unweighted Pair Group Method with the Arithmetic Average (UPGMA) for the representative core CAS protein, specifically CAS1, using the MUSCLE algorithm v3 and MEGA v12 [43], with default parameters consisting of a gap open penalty of −2.90, a gap extension penalty of 0.0, and clustering based on k-mer distance. Multiple sequence alignments and phylogenetic analyses were performed using Clustal X v2.1 (European Molecular Biology Laboratory, Heidelberg, Germany) with default settings: gap opening penalty of 10, gap extension penalty of 0.2, and the Gonnet protein weight matrix. Dendrograms were visualized using the NJ Plot application [44]. The distance matrix was calculated using the Jaccard coefficient. Conservation of CAS1 proteins was evaluated using the Geneious global alignment algorithm v2024.0 (Needleman–Wunsch) with a gap open penalty of −2.90 and a gap extension penalty of 0.0 under the default scoring matrix [45].

### 2.4. Determination of Conservation of Direct Repeats (DRs)

The conservation of direct repeats (DRs) was depicted using WebLogo 3.0 (University of California, Berkeley, CA, USA), which generated sequence logos (graphical representations of patterns in multiple sequence alignments) [46]. WebLogo employs PostScript code and algorithms derived from the alpro and makelogo programs, both part of Tom Schneider’s regulatory sequence analysis package written in python 3.7 [47]. The input for WebLogo consisted of multiple sequence alignments of DRs, which were generated using the MUSCLE algorithm v3 [43], with default parameters: a gap open penalty of −400, a gap extension penalty of 0, and clustering based on k-mer distance. While WebLogo provides a qualitative visualization of sequence conservation, quantitative conservation values were calculated separately. Specifically, average conservation was estimated as the mean frequency of the most common nucleotide at each position in these alignments of DRs identified with CRISPRFinder. This approach follows standard methodologies for evaluating conservation in repetitive elements within CRISPR-Cas systems [27].

### 2.5. Identification of Mutant Variables and Thermodynamics of Direct Repeats (DRs)

The prediction of RNA secondary structures of the CRISPR-type direct repeats (DRs), as well as the minimum free energy (MFE) of DRs and complete CRISPR arrays (including spacer sequences and DRs), was performed using the RNAfold WebServer (Institute for Theoretical Chemistry, University of Vienna, Vienna, Austria) (http://rna.tbi.univie.ac.at/cgi-bin/RNAWebSuite/RNAfold.cgi; accessed on 30 December 2023) [48]. The analysis was carried out using default parameters, which include prediction of the minimum free energy structure and partition function, application of the Turner 2004 energy model at 37 °C, no dangling ends, no constraint folding, and a maximum base pair span limited to the sequence length. The proposed criteria for predicting the secondary structures of the prokaryotic interference precursor small RNAs (siRNAs) transcribed by the CRISPR loci, based on DRs, were compared [45,49] The RNAfold program generated folding kinetics of DRs, where web server barriers allowed for the calculation of suboptimal structures within a predefined energy range. This provides insight into all the intermediate conformations of the RNA molecule before reaching the final structure. Additionally, the MFold program was used to determine the thermodynamic parameters of DRs, such as ΔG (Gibbs free energy), ΔH, ΔS, and Tm.

### 2.6. Identification of Antibiotic Resistance Genes in Bacteria and Archaea

Thirty-five genes ((AAC(6′)-Ic, AAC(6′)-Ie-APH(2′′)-Ia, AAC(6′)-Ii, AAC(6′)-Ip, AadA7, amp, APH(3′)-IIIa, ARR-2, blaCTX, CARB-2, cat-B, CblA-1, dfrA1, ErmB, fosa, fusB, mcr-1, meca, nimA, OXA, rmtD, sat-2, sat-4, SRT, sul1, TEM, tetC, tetL, tetM, teto, tetQ, tetW, tetX, VanR-A, and QnrA1) that confer resistance to different groups of antibiotics in Bacteria were selected. The genetic sequences of these genes were downloaded from GenBank and compared to the 60 complete bacterial genomes (30 genomes previously selected with confirmed CRISPR structures and 30 other genomes without CRISPR structures, according to CRISPRCas++) and 30 complete archaeal genomes (30 genomes studied with confirmed CRISPR structures) using the BLAST v2.15.0 to find homologous sequences with ≥85% identity and 100% query coverage (minimum of 28/33 matching nucleotides) [42,45]. A search for the plasmid sequences of the studied bacterial species was conducted.

### 2.7. Statistical Analysis

A one-way analysis of variance (ANOVA) and Pearson correlation analysis were employed as statistical methods when necessary or relevant to assess significant differences among the analyzed parameters and to evaluate the strength and direction of linear relationships between variables. The one-way ANOVA was applied to compare the means of multiple groups, determining whether observed differences were statistically significant, while Pearson correlation coefficients (r) were calculated to quantify the degree of association between continuous variables.

Statistical significance was established at a threshold of *p* < 0.05, indicating that differences or correlations observed were considered statistically significant when the probability of the result occurring by chance was less than 5%.

## 3. Results

### 3.1. CRISPR Structures Identification

Of the 30 archaeal genomes studied, 115 typical CRISPR arrays were found to be distributed with an average of 3-4 CRISPR loci per genome (30%, 9/30), and a minimum and maximum of 1 (16.66%, 5/30) and 10 (3.33%, 1/30), respectively (Table S1). According to their relative location on the chromosome, CRISPR loci were designated as CRISPR1…CRISPR10, corresponding to types I-A, I-B, I-D, I-E, I-U, III-A, III-B, III-C, and III-D of Class 1. The length of the CRISPR arrays in this study vastly varied from 108 bp (0.87%, 1/115) to 14,800 bp (0.87%, 1/115), with an average length of approximately 2012 bp. The number of spacers ranged from 1 (0.87%, 1/115) to 221 (0.87%, 1/115), with an average of 32 (0.87%, 1/115) per CRISPR structure, and spacer lengths averaged 41 bp (13%, 15/115) and were interspersed with direct repeats (DRs) ranging from 23 bp (6.1%, 7/115) to 44 bp (0.87%, 1/115) (Table S1). Most of the archaeal strains in this study were sourced from acidic hydrothermal sediments (22/30), with a smaller number isolated from human intestines (2/30), sludge (3/30), mines (1/30), rice rhizosphere (1), and Antarctica (1/30) (Table S1).

In total, 50 typical CRISPR arrays were found to be distributed among the 30 studied bacterial genomes, with an average of 1-2 CRISPR loci per genome (46.66%, 14/30), and a minimum and maximum of 1 (46.66%, 14/30) and 6 (3.33%, 1/30), respectively (Table S2). According to their relative location on the chromosome, CRISPR loci were designated as CRISPR1…CRISPR6. These correspond to types I-B, I-C, I-E, I-F, III-A, and III-D of Class 1, and types II-A and II-C of Class 2. The length of the CRISPR arrays in this study quite varied, but they were generally smaller than those found in Archaea, and ranged from 90 bp (2%, 1/50) to 4347 bp (2%, 1/50), with an average length of approximately 1077 bp. The number of spacers varied from 1 (2%, 1/50) to 72 (2%, 1/50), with an average of 16 per CRISPR structure (2%, 1/50). Spacer lengths averaged 31 bp (8%, 4/50) and were interspersed with DRs ranging from 28 bp (30%, 15/50) to 37 bp (8%, 4/50) (Table S2). The bacterial strains in this study mostly corresponded to clinical isolates (18/30), followed by animal isolates (5/30), environmental samples (4/30), food sources (2/30), and one strain of undetermined origin (Table S2).

The statistical analysis based on the *T-test* for two samples assuming unequal variances revealed significant differences between the CRISPR structures of Archaea and Bacteria. The mean number of confirmed CRISPR structures was 4 in Archaea and 2 in Bacteria. There was also a significant difference (*p* < 0.001) of 687 bp in the average length of the CRISPR arrays between the two domains, as well as a significant difference in the average number (*p* < 0.00007) of spacer sequences (16) and their lengths (7 bp).

### 3.2. Genes Associated with CRISPR and CAS Proteins

Twenty-nine putative *cas* genes were identified upstream and downstream of the CRISPR region in the studied genomes of Archaea and Bacteria. In all the genomes analyzed with the defined CRISPR structures, the genes associated with CRISPR were found, including core *cas* genes (*cas1*, *cas2*, *cas3*, *cas4*) as well as various subtype-specific genes belonging to the *csa*, *csb*, *csc*, *cse*, *csm*, *csn*, *csx*, *csy*, and *cmr* families. All the detected *cas* genes in Archaea belonged to Class 1, whereas they belonged to Classes 1 and 2 in Bacteria. In Archaea, these genes were distributed across various CRISPR subtypes, with a variable number of *cas* genes, as shown in Table 1.

In the Bacteria studied, the *cas* genes showed less variation compared to Archaea, which allowed the observation of the typical architectures of subtypes (Table 2).

The statistical analysis between *cas* genes showed no statistically significant difference (*p* > 0.01) between the number of *cas* genes in Archaea and the number of *cas* genes in Bacteria, with averages of 7 and 6, respectively. Therefore, a difference in one gene was found between the two studied domains.

**Figure 1:**
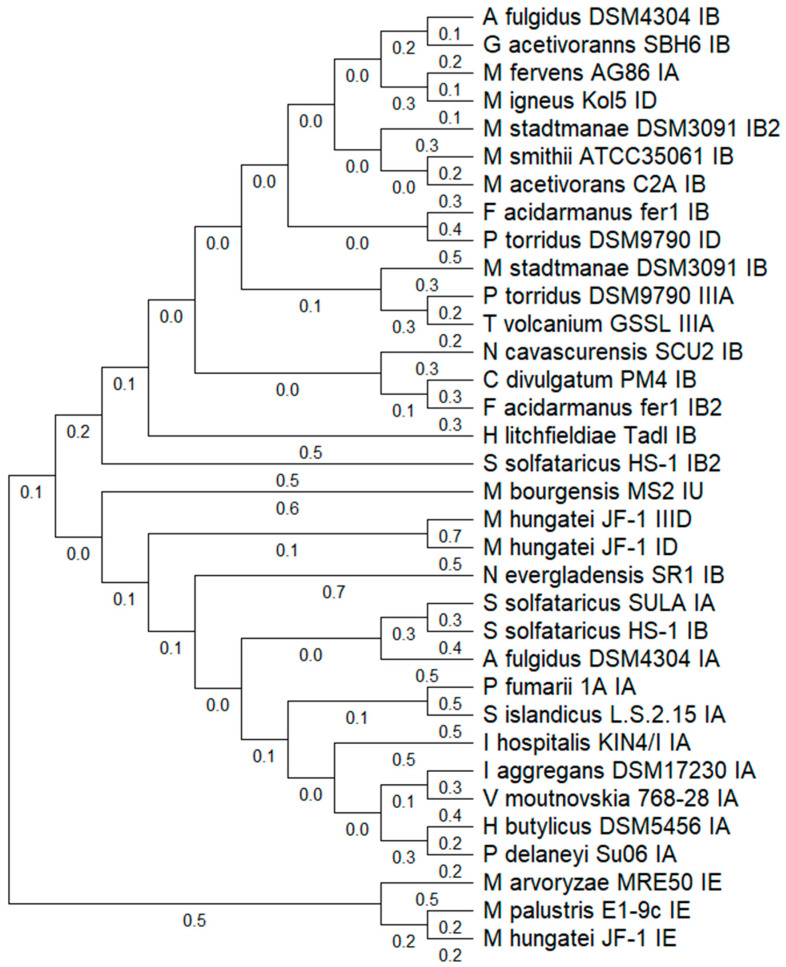
Phylogenetic tree of CAS1 proteins in archaeal genomes. The UPGMA tree of the CAS1 protein was generated using the MUSCLE algorithm in MEGA v12. Representative CAS1 proteins from all identified subtypes were selected. Evolutionary history was inferred using the *Neighbor-Joining* method. The optimal tree with the sum of branch length = 15.499 is shown (below the branches). The evolutionary distances were computed using the Poisson correction method and are in the units of the number of amino acid substitutions per site. The analytical procedure encompassed 34 amino acid sequences. The pairwise deletion option was applied to all ambiguous positions for each sequence pair, resulting in a final data set comprising 596 positions.

The phylogenetic relationship is based on the homology of Cas1 proteins that group Archaea species, in several cases, according to their CRISPR-Cas subtypes: IA, IB, ID, IE, IU, IIIA, and IIID (Figure 1). Notable variability in Cas1 proteins was observed within Archaea in terms of subtype organization, evidenced by the distinct phylogenetic distances among Cas1 proteins classified under the same subtype, such as IB, and their relative proximity to proteins from other subtypes, such as IIIA or IA. This dispersion suggests events of functional divergence or horizontal gene transfer.

Subtype IA exhibited higher conservation among species, clustering into a clade with short phylogenetic distances, which may indicate a lower rate of evolutionary divergence. In contrast, Cas1 proteins from subtype IE showed the greatest phylogenetic distances observed, indicating high structural and possibly functional variability. Multiple Cas1 protein copies were also detected in certain species, such as *Methanocaldococcus hungatei* JF-1, suggesting the presence of more than one CRISPR-Cas locus in their genome (Figure 1).

**Figure 2:**
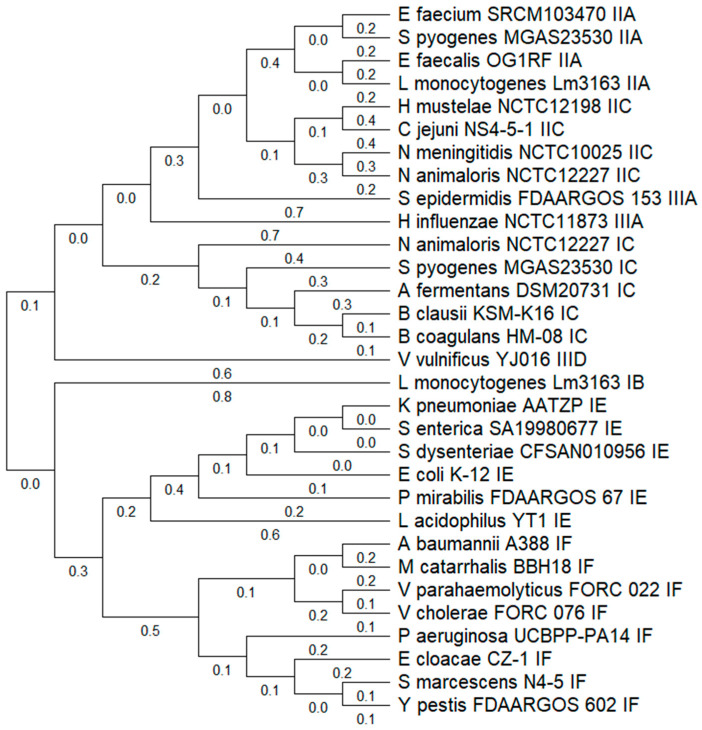
Phylogenetic tree of CAS1 proteins in bacterial genomes. The UPGMA tree of the CAS1 protein was generated using the MUSCLE algorithm in MEGA v12. Representative CAS1 proteins from all identified subtypes were selected. Evolutionary history was inferred using the *Neighbor-Joining* method. The optimal tree with the sum of branch length = 11.959 is shown (below the branches). The evolutionary distances were computed using the Poisson correction method and are in the units of the number of amino acid substitutions per site. The analytical procedure encompassed 31 amino acid sequences. The pairwise deletion option was applied to all ambiguous positions for each sequence pair, resulting in a final data set comprising 759 positions.

The phylogenetic relationship based on Cas1 protein homology grouped the bacterial species according to their CRISPR-Cas subtypes, including IB, IC, IIA, IIC, IIIA, IIID, IE, and IF (Figure 2). Similarly to Archaea, a notable variability in Cas1 protein sequences was observed, with some proteins from the same subtype (e.g., IC or IE) displaying substantial phylogenetic distances. This pattern suggests functional divergence or potential horizontal gene transfer events within Bacteria.

Subtype IIA showed a relatively conserved grouping, with strains such as *Enterococcus faecium*, *E. faecalis*, and *Streptococcus pyogenes* clustering closely together. In contrast, subtype IE presented the broadest distribution, with large phylogenetic distances among species like *E. coli*, *Salmonella enterica*, and *Shigella dysenteriae*, pointing to high structural and possibly functional diversity within this group.

Several subtypes were shared across diverse genera, such as subtype IF, which was found in both *Pseudomonas aeruginosa* and *Vibrio cholerae*, suggesting convergent evolution or acquisition through horizontal gene transfer. Additionally, some species, *like Listeria monocytogenes*, appeared in more than one CRISPR-Cas subtype group (IB and IIC), indicating the presence of multiple Cas1 loci in their genomes (Figure 2).

The relation of the CAS1 proteins between Archaea and Bacteria regrouped them according to the different CRISPR subtypes and formed diverse phylogenetic groups, where the shortest distance corresponded to CAS1 of subtype IC. Similarly, the homogeneity of these proteins was noted for subtypes IA, IE, and IF, represented by four well-defined groups (Figure S1). At a longer distance were the CAS1 proteins of subtypes IIC and IIC, which formed two closely related groups due to the architectural characteristics of the type II CRISPRs found exclusively in the studied Bacteria. The CAS1 proteins of subtype IB were, in this case, observed to be more closely related, and formed two groups separated by the CAS1 of subtypes IA and ID (Figure S1).

**Figure 3:**
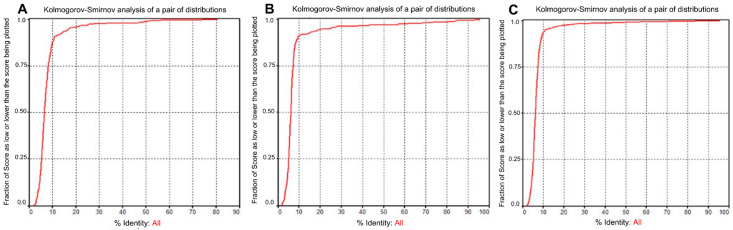
Comparison of CAS1 proteins in archaeal and bacterial genomes. (**A**) Percentage identity of CAS1 proteins in the archaeal genomes analyzed. (**B**) Percentage identity of CAS1 proteins in the bacterial genomes analyzed. (**C**) Percentage identity of CAS1 proteins in the analyzed archaeal and bacterial genomes. The cumulative curves show the fraction of CAS1 protein sequences with a percentage identity equal to or lower than the value indicated on the X-axis. The red line represents the cumulative distribution of sequence identities.

The Kolmogorov–Smirnov pairwise distribution analysis (Figure 3A–C) showed the frequency and identity of the CAS1 proteins from the Archaea and Bacteria studied. Although there was a slight difference in these variables between the two domains, Archaea had a higher degree of homology (the CAS1 with ≥60% homology had appearance frequencies of >99%) (Figure 3A) than the CAS1 of Bacteria (CAS1 with ≥85% homology and had appearance frequencies of >99%) (Figure 3B). Hence the frequency of the CAS1 polymorphisms was high in Bacteria with ranges of 0.8–1.0 for the homologies of ≥10%. In Archaea, ranges were 0.9–1.0 for homologies of ≥10%. Figure 3C shows the high degree of homology and identity between the CAS1 from Archaea and Bacteria, and it also indicates a high frequency of polymorphisms (most of which were between 95–99%).

### 3.3. Determination of the Conservation of Direct Repeats (DRs)

**Figure 4:**
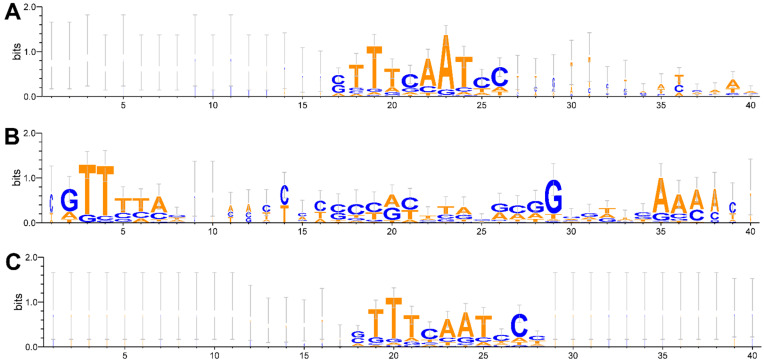
Conservation of direct repeats (DRs). The sequence logo was created using WebLogo 3.7.4. (**A**) Conservation of DRs in archaeal genomes. (**B**) Conservation of DRs in bacterial genomes. (**C**) Conservation of DRs in archaeal and bacterial genomes. Error bars are shown in gray.

In Archaea, CRISPR arrays with typical direct repeats (DRs) were found, with a minimum number of 2 and a maximum of 222 DR, whose lengths were between 23 and 44 bp (Table S1). The strain that presented the smallest CRISPR loci was Mac.C2A, with a size of 2 DRs located in CRISPR4, while the longest CRISPR loci from the Sa.HS-1 strain, which had 222 DRs located in CRISPR1 of its genome. The CRISPR were compared using multiple sequence alignments. The comparative analysis between the DRs showed a significant degree of conservation of the repeated sequences with 94.72%, calculated based on the multiple sequence alignments. This was found despite the existence of the non-consensus repeats characterized by point mutations in various regions of these sequences within each evaluated CRISPR array. The conservation of DRs shown by WebLogo v3.7.4 indicated a highly conserved region in all the CRISPR loci of the analyzed Archaea genomes, located between bp 13 and 22 of this sequence (Figure 4A).

In Bacteria, CRISPR arrays with typical DRs were found, with a minimum number of 2 and a maximum of 73 DRs, whose length was between 28 and 37 bp (Table S2). The strain with the smallest CRISPR loci in this case was Sd.CFSAN010956, whose size was 2 DRs located in its only CRISPR, while the longest CRISPR loci were from the Vc.FORC_076 strain with 73 DRs also located in the only CRISPR present in its genome. The comparative analysis among DRs demonstrated substantial sequence conservation, with an average similarity of 93.52%, calculated based on the multiple sequence alignments. This was found despite the existence of the non-consensus repeats characterized by point mutations in various regions of these sequences in each evaluated CRISPR array. The conservation of DRs shown by WebLogo 3.7.4 once again indicated the degree of conservation located between bp 3 and 7 and between bp 14 and 39 of the sequences (Figure 4B), being more homogeneous than in Archaea.

The combined WebLogo (Figure 4C), representing both archaeal and bacterial DRs, reveals a consensus pattern that retains key conserved elements from each group (especially the central TTTCAATCC motif seen in Archaea), yet shows reduced overall conservation compared to the archaeal-only alignment. This indicates that, while some core features are shared, domain-specific sequence variations persist.

### 3.4. Identification of the Mutant Variables and Thermodynamics of Direct Repeats (DRs)

When predicting the secondary structure of prokaryotic precursor species of RNA, crucial in the adaptive immunity mechanism, it was found that, on average, all the DRs from the studied Archaea CRISPR loci could form an RNA secondary structure with a thermodynamic ensemble free energy or minimum free energy (MFE) of −3.66 kcal/mol (0.87% or 1/115), with a minimum of −0.05 kcal/mol (0.87% or 1/115) and a maximum of −13.36 kcal/mol (1.74% or 2/115) (Table S3 and Figure S2). In contrast, the formation energy of the transcribed CRISPR-like structures obtained by calculating the MFE from the complete structure (DRs plus spacers) showed that the complete CRISPR structures had an average of −557.23 kcal/mol, with a minimum of −54.16 kcal/mol (0.87% or 1/115) and a maximum of −2908.89 kcal/mol (0.87% or 1/115) (Table S3, Figure S4). The non-consensus DRs that presented point mutations distributed along typical sequences were also evaluated to predict the secondary structure of these variant DRs. The average MFE of the non-consensus DRs was −3.37 kcal/mol (0.81% or 1/123), with a minimum of −0.03 kcal/mol (0.81% or 1/123) and a maximum of −13.41 kcal/mol (0.81% or 1/123) (Table S3). Based on this, no statistically significant difference (*p* > 0.01) appeared in the MFE between the consensus and mutated DRs from Archaea CRISPR loci. In 86.7% (26/30) of the analyzed Archaea genomes, variant or mutant DRs were obtained in CRISPRs, while the remaining 13.3% (4/30) corresponded only to species Fa.fer1, Ms.ATCC35061, Ne.SR1, and Sa.HS-1 (Table S3). CRISPR can exist in various conformations in nature and eventually reach the most thermodynamically stable form (Figure S2). Therefore, when analyzing the folding kinetics of the Archaea DRs, an average of 5465.75 thermodynamically probable conformational forms were obtained, with a minimum of 10 and a maximum of 414,089 possible structures (Table S3).

Similarly in Bacteria, on average, all the DRs from CRISPR loci could form an RNA secondary structure with a thermodynamic ensemble free energy or MFE of −9.00 kcal/mol (2% or 1/50), with a minimum of −0.45 kcal/mol (2% or 1/50) and a maximum of −16.49 kcal/mol (2% or 1/50) (Table S4 and Figure S3). In contrast, the formation energy of the transcribed CRISPR-like structures obtained by calculating the MFE from the complete structure (DRs plus spacers) showed that the complete CRISPR structures had an average of −426.69 kcal/mol, with a minimum of −90.83 kcal/mol (2% or 1/50) and a maximum of −2083.90 kcal/mol (2% or 1/50) (Table S4, Figure S5). The non-consensus DRs that presented point mutations distributed along typical sequences were also evaluated to predict the secondary structure of these variant DRs. The average MFE of the non-consensus DRs was −8.28 kcal/mol (1.64% or 1/61), with a minimum of −0.42 kcal/mol (1.64% or 1/61) and a maximum of −15.69 kcal/mol (1.64% or 1/61) (Table S4). Similarly to Archaea, there was no statistically significant difference (*p* > 0.01) in the MFE between the consensus and the mutated DRs from the Bacteria CRISPR loci.

As with Archaea, in 86.7% (26/30) of the analyzed genomes of Bacteria, variant or mutant DRs were obtained in the CRISPR loci, while the remaining 13.3% (4/30) corresponded only to species Ab.A388, Hm.NCTC12198, Sd.CFSAN010956, and Na.NCTC12227 (Table S4). When analyzing the folding kinetics of the Bacteria DRs, an average of 6843.41 thermodynamically probable conformational forms were obtained, with a minimum of 214 and a maximum of 59998 possible structures (Table S4).

**Figure 5:**
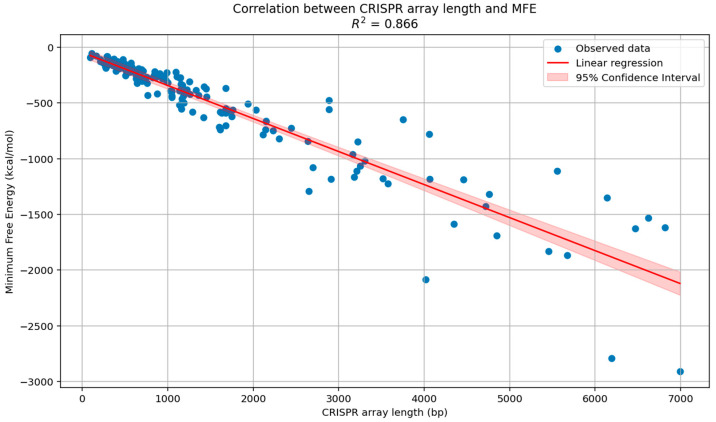
Correlation between the length (bp) of the CRISPR arrays and the minimum free energy (MFE) of assembly of the CRISPR loci found in Archaea and Bacteria. A very strong positive correlation was observed (R^2^ = 0.87).

Statistical analyses indicated a very high correlation (R^2^ = 0.87) between the size of CRISPR loci (in base pairs) and the MFE of the ensemble (DRs plus spacer sequences) in both Archaea and Bacteria. This suggests that as CRISPR length grew, the MFE of formation also increased, which makes the assembly of the RNA secondary structure more favorable due to exothermic processes (energy release) (Figure 5). A low correlation (R^2^ = 0.20) was found between the MFE and folding kinetics of the DRs in the CRISPR loci in Archaea, while a moderate correlation (R^2^ = 0.47) was found for the same variables in Bacteria. Hence, there was no statistically significant evidence (*p* > 0.01) to indicate that a higher or a lower MFE of DRs would lead to greater or lesser conformations or secondary RNA structures. Although there was no significant difference between the averages of the MFE for the consensus and mutated DRs in Archaea, similar to Bacteria, significant differences (*p* < 0.01) were found when comparing the MFE of the consensus DRs between Archaea (−3.66 kcal/mol) and Bacteria (−9.00 kcal/mol). Similarly, there was a significant difference (*p* < 0.01) for the DRs presenting mutant variants in both Archaea and Bacteria, with averages of −3.37 kcal/mol and −8.29 kcal/mol, respectively (Figures S2 and S3). There was no significant evidence (*p* > 0.01) between the average MFE of the CRISPRs in Archaea and Bacteria.

When comparing thermodynamic parameters, a significant statistical difference (*p* < 0.01) appeared in the enthalpy (ΔH), entropy (ΔS), and melting temperature (Tm) of the DRs between Archaea and Bacteria. There was more energy in the molecular system (RNA secondary structures of DRs) in Bacteria (−75.73 kcal/mol) than in Archaea (−48.76 kcal/mol), which indicates less heat generation in the latter domain. As enthalpy is directly proportional to entropy, there was a greater molecular equilibrium or organization for the CRISPR loci in Bacteria (−214.63 cal/(K·mol)) compared to Archaea (−145.49 cal/(K·mol)). However, in both domains, exothermic reactions dominated as ΔH < 0.

### 3.5. Antibiotic Resistance Genes in Bacteria and Archaea

When comparing each genome of the studied Archaea species (without CRISPR structures and with confirmed CRISPR structures) and Bacteria with reference sequences of antibiotic resistance genes, in Archaea, 30% of the species (9/30) had at least one antibiotic resistance gene in their genome (Figure S6). Of the Bacteria species with confirmed CRISPR structures, 46.67% (14/30) showed antibiotic resistance genes (Figure S7). In Bacteria without the presence of CRISPR-Cas systems, the genomes of 63.33% of species (19/30) harbored resistance genes (Figure S8). Most Bacteria species without confirmed CRISPR-Cas systems were isolated from human clinical sources, which accounted for 40% (12/30), followed by animal origin with 16.7% (5/30), human microbiota with 10% (3/30), soil isolates with 10% (3/30), food isolates with 6.7% (2/30), and marine, air, wastewater, bioreactor and vegetable isolates, each with 3.3% (1/30) (Table 3).

The bacterial strains in this study with confirmed CRISPR-Cas systems were mostly from clinical isolates and comprised 60% (18/30), followed by animal isolates with 16.67% (5/30), environmental sources with 13.33% (4/30), food sources with 6.67% (2/30), and one strain of undetermined origin with 3.33% (1/30) (Table S2). In contrast, most of the Archaea strains in this study originated from acidic hydrothermal sediments, and accounted for 73.33% (22/30), along with human gut isolates with 6.66% (2/30), mud with 10% (3/30), mines with 3.33% (1/30), rice rhizosphere with 3.33% (1/30), and Antarctica with 3.33% (1/30) (Table S1).

Based on the analysis of antibiotic resistance genes (ARGs), only three types—nimA, tetC, and AAC(6′)-Ie-APH(2′′)-Ia—were identified in Archaea, appearing in different combinations across 9 of the 30 strains studied (Figure S6). Among the 15 gene occurrences detected in Archaea, 46.67% (7/15) were from species isolated from thermal waters, 26.67% (4/15) from human microbiota, 13.33% (2/15) from marine sediments, and 6.67% (1/15 each) from acidic substrates and volcanic soils.

In contrast, 32 ARGs were identified in bacterial strains with confirmed CRISPR-Cas systems, the majority of which (84.38%, or 27/32) were found in clinical isolates. The remaining genes were distributed across environmental (6.25%), undetermined (6.25%), and food-related sources (3.13%). In Bacteria lacking confirmed CRISPR-Cas systems, the number of ARGs increased to 42, mostly found in clinical strains (45.24%). The rest were found in strains from animals (14.29%), bioreactors (9.52%), soil (7.14%), air (7.14%), wastewater (4.76%), food (4.76%), marine samples (2.38%), plants (2.38%), and human microbiota (2.38%). Overall, 51.69% (46/89) of all ARGs were identified in clinical isolates across both domains.

Among Archaea, the species with the fewest ARGs (two per genome) were *Ignisphaera aggregans*, *Methanocaldococcus fervens*, *Methanotorris igneus*, *Methanobrevibacter smithii*, and *Methanosphaera stadtmanae*. In Bacteria with CRISPR-Cas, Acinetobacter baumannii A388 had the highest number (six genes), while Streptococcus pneumoniae NT11058 led the group without CRISPR, with seven ARGs. Notably, within the same species, differences emerged: A. baumannii A388 (CRISPR-positive) carried six genes, while A. baumannii 11510 (CRISPR-negative) had only two. Similarly, Pseudomonas aeruginosa Y71 (no CRISPR) had six ARGs, compared to five in P. aeruginosa UCBPP-PA14 (with CRISPR).

From the 35 unique ARGs detected, 24 (68.57%) appeared across both Archaea and Bacteria. These included genes conferring resistance to aminoglycosides, beta-lactams, tetracyclines, macrolides, colistin, nitroimidazoles, fosfomycin, phenicols, sulfonamides, and streptomycin (Figures S6–S8). Resistance to aminoglycosides, beta-lactams, and tetracyclines represented 25% each of this shared pool, while other types occurred at 4.17% each.

In total, 89 ARG occurrences were recorded: 42 (47.19%) in Bacteria without CRISPR, 32 (35.96%) in Bacteria with CRISPR, and 15 (16.85%) in Archaea. Within Archaea, the three detected genes conferred resistance to tetracyclines (13.33%), aminoglycosides (40%), and nitroimidazoles (46.67%).

Some ARGs were exclusive to Bacteria without CRISPR (e.g., APH(3′)-IIIa, blaCTX, ErmB, sat-4, tetL, tetM, tetO, tetQ, tetW). Others—like AadA7 and mcr-1—were exclusive to CRISPR-positive strains. Several genes (amp, mecA, OXA, TEM, sul1) appeared equally in both groups. Additionally, genes such as cat-B, SRT, rmtD, AAC(6′)-Ie, APH(2′′)-Ia, nimA, AAC(6′)-Ic, and fosA were found in both bacterial groups, with varying frequencies.

Lastly, plasmids were detected in 21.11% (19/90) of all analyzed species. Among them, 8/30 (26.67%) were in Bacteria without CRISPR, 10/30 (33.33%) in Bacteria with CRISPR, and only 1/30 (3.33%) in Archaea. In total, 34 plasmids were identified: 47.06% in CRISPR-negative Bacteria, 50% in CRISPR-positive Bacteria, and 2.94% in Archaea (Tables S5–S7).

## 4. Discussion

### 4.1. Structural and Functional Differences Between Archaea and Bacteria

The results reveal significant differences in the quantity, length, and complexity of CRISPR structures between Archaea and Bacteria. On average, archaeal genomes contain a greater number of CRISPR loci, as well as longer arrays with a higher number of spacers compared to bacterial genomes. This increased complexity may be associated with the exposure of Archaea to extreme environments and greater viral infection pressure. The presence of multiple CRISPR system subtypes within a single genome—especially in Archaea—suggests a case of convergent evolution aimed at responding to a wide diversity of genetic threats. In contrast, the lower subtype diversity observed in Bacteria may reflect adaptation to more specific selective pressures, such as antibiotic use and environmental factors, which may have favored the adoption of alternative defense mechanisms [50]. These functional differences could be key to understanding why Archaea tend to maintain more complex CRISPR systems, while Bacteria have diversified their defensive strategies. These findings are consistent with recent studies that highlight the influence of ecological and evolutionary factors on the diversity of CRISPR-Cas systems. In particular, the work by Wang et al., 2023 demonstrates that in thermophilic Bacteria, the prevalence of CRISPR loci and subtype diversity increases with adaptation to extreme environments—a pattern also observed in the archaeal species analyzed in this study [23]. Similarly, Ghaffarian and Panahi 2024 reported considerable variability in the presence and types of CRISPR-Cas systems within the Acetobacter genus, emphasizing how different bacterial species retain or lose these systems depending on environmental selective pressures [51]. On the other hand, the comprehensive analysis of marine ecosystems by Li et al., 2024 showed that Archaea tend to possess more complex CRISPR systems with a greater number of subtypes, whereas many Bacteria—especially those with more compact genomes or associated with symbiotic lifestyles—often lack CRISPR-Cas systems or rely on alternative mechanisms such as restriction–modification systems [52].

### 4.2. Subtype Distribution and Evolutionary Implications

The high prevalence of CRISPR-Cas systems in Archaea contrasts with their lower representation in Bacteria, supporting the hypothesis that archaeal organisms rely more heavily on this adaptive immune mechanism [50]. The typical CRISPR matrices detected in Bacteria were consistent with those reported by Burstein et al. in 2016 and Liu and Doudna in 2020, showing a dominance of Class 1 systems, which are the most abundant in nature [53,54]. However, the distribution of subtypes was highly diverse. These findings differ from those reported by Makarova in 2015, who found subtype I-B to be the most abundant in Bacteria [25]. Our results instead showed a higher frequency of subtypes I-F, I-E, and I-C. The notable presence of subtype I-F highlights its potential importance in bacterial adaptive immunity and suggests that subtype prevalence may vary considerably depending on the sampled species and environmental contexts.

The dendrograms, where subtype clusters appear relatively distant from each other, illustrate the accuracy of the CRISPR database queries. These results were derived from research that initially identified CRISPR types based on direct repeat (DR) families rather than *cas* gene arrangement, further highlighting the close relationship between these components [37].

Additionally, we observed dispersion in the DRs associated with subtype I-B, indicating a link between DR families and the arrangement of their respective *cas* genes—subtype I-B being one of the subtypes with the greatest variation in *cas* gene architecture [37].

### 4.3. Genetic Architecture of Cas Genes

The variability observed in *cas* genes between Archaea and Bacteria is also notable. In Archaea, all identified *cas* genes belong exclusively to Class 1, whereas in Bacteria, both Class 1 and Class 2 genes were found. Moreover, the genetic architectures of CRISPR subtypes were more diverse in Archaea, which may reflect a greater evolutionary need to modulate immune responses against a broader range of genetic threats. This finding aligns with studies highlighting the complexity of the CRISPR-Cas system in extremophile organisms [27].

### 4.4. Role in Horizontal Gene Transfer and Antibiotic Resistance

An important aspect is the role of the CRISPR-Cas system in interfering with horizontal gene transfer (HGT). The data suggests that this system not only protects against phages but also regulates the incorporation of foreign genetic material, including antibiotic resistance genes [55,56]. This supports earlier studies showing how CRISPR-Cas can limit the spread of non-essential or potentially harmful genes [28]. However, the lower number of CRISPR loci in Bacteria may indicate a reduced dependence on this mechanism in favor of alternative defenses, such as restriction–modification systems.

### 4.5. Thermodynamic Stability of Direct Repeats

The biological and functional relevance of thermodynamic parameters, such as minimum free energy (MFE), enthalpy, entropy, and melting temperature (Tm), is fundamental to the specificity and efficiency of CRISPR-Cas systems. MFE determines the most stable secondary structure of the sgRNA, which is essential for adopting the correct conformation and effectively binding to the Cas protein, enabling precise recognition of the target DNA and minimizing mismatch tolerance [57].

The formation of the Cas–sgRNA complex is primarily driven by entropy changes associated with water molecule release and conformational rearrangement, whereas the interaction between the complex and the target DNA is enthalpically favored, due to the formation of hydrogen bonds and base stacking within the DNA–RNA hybrid [57,58].

Additionally, the Tm of the sgRNA–DNA duplex is a direct indicator of binding stability: a high Tm reflects strong and specific interactions, while the presence of mismatches in off-target sequences lowers the Tm, thereby reducing the likelihood of unintended cleavage [58,59].

These parameters influence not only binding affinity but also the energetic barriers that regulate the transition from recognition to cleavage. As a result, only sgRNA–DNA combinations with optimal thermodynamic profiles enable efficient and specific genome editing [59,60]. Therefore, integrating MFE, enthalpy, entropy, and Tm into the rational design of sgRNAs is key to maximizing specificity and minimizing off-target effects in genome editing applications.

The results based on the minimum free energy (MFE) of consensus and mutated DRs in Archaea and Bacteria revealed significant differences between domains but not within sequences of the same domain. This indicates that the sporadic mutations found across various regions of the repeat sequences did not lead to notable changes in the thermodynamic average of MFE for typical DRs and their variants. However, structural variation was evident between the domains. DRs in Archaea exhibited less stable RNA secondary structures compared to those in Bacteria, which showed higher MFE values. To validate these computational predictions, we now propose experimental approaches such as SHAPE (Selective 2′-Hydroxyl Acylation analyzed by Primer Extension), RNase structure probing, or thermal denaturation assays. These techniques would allow direct measurement of RNA structure stability and folding dynamics of archaeal and bacterial DRs, thus providing empirical support to the thermodynamic differences inferred from MFE calculations.

Previous studies have shown that RNA structures with longer stems tend to have lower MFE and greater stability [49], highlighting the importance of stem length in structural thermodynamics. Accordingly, the shorter stems observed in archaeal DRs result in less thermodynamic stability compared to the longer, more stable stems of bacterial DRs.

Some authors emphasize the importance of analyzing complete CRISPR units (including both DRs and spacers) to determine which structure is thermodynamically more stable—not just based on the MFE of DRs, but also considering the MFE of spacers [49]. In this study, the average MFE of complete CRISPR arrays showed no significant difference between Archaea and Bacteria.

### 4.6. Environmental Influence and Species-Specific Factors

The presence of antibiotic resistance genes in Bacteria has been shown to depend on both environmental factors and species-specific traits, as evidenced by recent studies. The interaction between external factors—such as antibiotic and nutrient contamination—and intrinsic bacterial characteristics (e.g., efflux pump systems or horizontal gene transfer capabilities) influences the prevalence and distribution of these resistance genes [61,62]. The environment modulates gene expression in a species-dependent manner. For instance, high microbial density environments, like wastewater, promote horizontal gene transfer via plasmids, but the efficiency of this process varies by species, depending on their natural competence to acquire exogenous DNA [62].

### 4.7. Future Perspectives

One of the main limitations of this study is the heterogeneity of the bacterial strains included in the analysis. Although this diversity allowed us to explore broad interspecies patterns in the distribution of CRISPR-Cas systems and antibiotic resistance genes (ARGs), it may have limited our ability to detect statistically significant differences in ARG content between CRISPR-positive and CRISPR-negative groups. The strains varied widely in taxonomy and ecological origin, which introduces confounding factors such as host-associated selective pressures, environmental exposure, and genome size. Future studies focusing on strains of the same bacterial species, ideally isolated from similar clinical or environmental contexts, would help control these variables and provide a more refined understanding of the relationship between CRISPR systems and antimicrobial resistance.

While this study provides a detailed analysis of the distribution and genetic diversity of CRISPR-Cas systems, further sampling is necessary to include more archaeal and bacterial genomes from diverse habitats. Moreover, experimental exploration would be valuable to assess how these differences affect CRISPR-Cas functionality in response to specific genetic threats. Finally, additional comparative studies could help clarify why certain subtypes predominate in specific environments or evolutionary lineages.

## 5. Conclusions

This study provides a comparative genomic and structural analysis of CRISPR-Cas systems and antibiotic resistance genes (ARGs) in Archaea and Bacteria. Our findings highlight several key differences between these domains. First, Archaea exhibit greater structural complexity and diversity in CRISPR-Cas systems, including multiple subtypes per genome and consistently longer CRISPR arrays. This suggests an evolutionary adaptation to extreme environments and a reliance on CRISPR-mediated immunity. In contrast, Bacteria displayed fewer CRISPR loci, a wider range of CRISPR-Cas classes (including Class 2 systems), and greater variability in subtype distribution, indicating alternative evolutionary strategies for genetic defense.

Second, thermodynamic analyses revealed that bacterial direct repeats (DRs) tend to form more stable RNA secondary structures than those in Archaea, with significantly lower minimum free energy (MFE) values, higher enthalpy, and melting temperatures. These differences may reflect the structural requirements for Cas–RNA complex formation and efficiency in each domain.

Third, an inverse relationship was observed between the presence of CRISPR-Cas systems and the abundance of ARGs in bacterial genomes. Bacteria lacking CRISPR systems harbored a greater number and diversity of resistance genes, supporting the hypothesis that CRISPR-Cas may limit horizontal gene transfer and acquisition of exogenous elements such as plasmid-borne ARGs.

Together, these results underscore the evolutionary divergence and ecological significance of CRISPR-Cas systems. They not only function as adaptive immune tools but may also serve as modulators of genomic plasticity. Future studies using species-specific sampling and experimental validation of thermodynamic predictions will help to refine our understanding of the functional interplay between CRISPR systems and antibiotic resistance.

## Figures and Tables

**Table 1:** Distribution of CRISPR-associated genes in Archea.

Subtype	Clase	Associated Genes	Number of Arrays	Strains
**IA**	I	*cas1-7*, *csa5*, *csaX*	10	*Ferroglobus placidus DSM_10642*, *Aeropyrum pernix K1*, *Ignisphaera aggregans DSM17230*, *Pyrodictium delaneyi Su06*, *Saccharolobus solfataricus SULA*, Archaeoglobus fulgidus DSM_4304 *, Ignicoccus hospitalis KIN4/I *, Hyperthermus butylicus DSM5456 *, Pyrolobus fumarii 1A *, Thermoproteus tenax Kra1 *, Sulfodiicoccus acidiphilus HS-1 *, Sulfolobus islandicus L.S.2.1.5 *, Vulcanisaeta moutnovskia 768-28 *, Methanotorris igneus Kol5 *, Methanocaldococcus fervens AG86 *
**IB**	I	*cas1-8*	7	*Geoglobus acetivorans SBH6*, *Cuniculiplasma divulgatum PM4*, *Methanobrevibacter smithii ATCC35061*, *Halohasta litchfieldiae tADL*, *Nitrososphaera evergladensis SR1*, *Nitrosocaldus cavascurensis SCU2*, *Ferroplasma acidarmanus Fer1*, *Methanosphaera stadtmanae DSM_3091*, Sulfodiicoccus acidiphilus HS-1 *, Methanosarcina acetivorans C2A *, Methanocaldococcus fervens AG86 *
**ID**	I	*cas1-4*, *cas6*, *cas10*, *csc1-2*	3	Methanosphaerula palustris E1-9c *, Pycrophilus torridus DSM9790 *, Methanospirillum hungatei JF-1 *, Sulfodiicoccus acidiphilus HS-1 *, Methanospirillum hungatei JF-1 *
**IE**	I	*cas1-3*, *cas5-7*, *cse1-2*	3	*Methanocella arvoryzae MRE50*, Methanosphaerula palustris E1-9c *, Methanospirillum hungatei JF-1 *
**IU**	I	*cas1*, *cas3*, *csb1-2*, *csx17*	1	*Methanoculleus bourgensis MS2*
**IIIA**	I	*cas1-2*, *cas6*, *cas10*, *csm2-5*	3	*Thermoplasma volcanium GSS1*, Methanosarcina acetivorans C2A *, Pycrophilus torridus DSM9790 *, Vulcanisaeta moutnovskia 768-28 *, Methanotorris igneus Kol5 *, Methanocaldococcus fervens AG86 *
**IIIB**	I	*cas6*, *cas10*, *cmr1*, *cmr3-6*, *csm3*	6	Archaeoglobus fulgidus DSM_4304 *, Ignicoccus hospitalis KIN4/I *, Hyperthermus butylicus DSM5456 *, Pyrolobus fumarii 1A *, Sulfolobus islandicus L.S.2.1.5*, Vulcanisaeta moutnovskia 768-28 *
**IIIC**	I	*cas10*, *cmr1*, *cmr3-6*	1	Methanocaldococcus fervens AG86 *
**IIID**	I	*cas1-2*, *cas4*, *cas6*, *csm3-4*	3	Thermoproteus tenax Kra1 *, Methanospirillum hungatei JF-1 *, Sulfodiicoccus acidiphilus HS-1 *, Sulfolobus islandicus L.S.2.1.5 *, Methanotorris igneus Kol5 *

Strains marked with * have multiple CRISPR subtypes (co-occurrence). Some strains appear under several subtypes due to this co-occurrence. In cases where multiple CRISPR-Cas subtypes co-occurred within the same genome, each subtype was treated as an independent observation for the purpose of subtype-level analyses.

**Table 2:** Distribution of CRISPR-associated genes in Bacteria.

Subtype	Clase	Associated Genes	Number of Arrays	Strains
**IF**	I	*cas1*, *cas2-cas3*, *cas6*, *csy1-3*	1	*Acinetobacter baumannii A388*, *Pseudomonas aeruginosa UCBPP-PA14*, *Serratia marcescens N4-5*, *Enterobacter cloacae CZ-1*, *Vibrio parahaemolyticus FORC_022*, *Vibrio cholerae FORC_076*, *Moraxella catarrhalis BBH18*, *Yersinia pestis FDAARGOS_602*
**IE**	I	*Variant 1: cas1-3*, *cas5-7*, *cse1-2; Variant 2: cas1-3*, *cas5-7*, *cse2*	2	*Escherichia coli K-12*, *Proteus mirabilis FDAARGOS_67*, *Klebsiella pneumoniae AATZP*, *Salmonella enterica SA19980677*, *Shigella dys-enteriae CFSAN010956*, *Lactobacillus acidophilus YT1*
**IC**	I	*cas1-5*, *cas7-8*	1	*Bacillus clausii KSM-K16*, *Acidaminococcus fermentans DSM20731*, *Bacillus coagulans HM-08*, Streptococcus pyogenes MGAS23530 *, Neisseria animaloris NCTC12227 *
**IIA**	II	*cas1-2*, *cas9*	1	*Enterococcus faecium SRCM103470*, *Enterococcus faecalis OG1RF*, *Listeria innocua Clip11262*, Listeria monocytogenes Lm3163 *, Streptococcus pyogenes MGAS23530 *
**IIC**	II	*cas1-2*, *cas9; csn2*	1	*Helicobacter mustelae NCTC12198*, *Campylobacter jejuni NS4-5-1*, *Neisseria meningitidis NCTC10025*, *Mycoplasma phocicerebrale 1049*, Neisseria animaloris NCTC12227 *
**IIIA**	I	*cas1-2*, *cas6*, *cas10*, *csm2-4*, *csm6*	1	*Staphylococcus epidermidis FDAARGOS_153*, *Haemophilus influenzae NCTC11873*
**IB**	I	*cas1-8*	1	Listeria monocytogenes Lm3163 *
**IIID**	I	*cas1-2*, *cas10*, *csm3*	1	*Vibrio vulnificus YJ016*

Strains marked with * have multiple CRISPR subtypes (co-occurrence). Some strains appear under several subtypes due to this co-occurrence. In cases where multiple CRISPR-Cas subtypes co-occurred within the same genome, each subtype was treated as an independent observation for the purpose of subtype-level analyses.

**Table 3:** Bacterial species (genomes) lacking CRISPR-Cas systems.

		Ref.
Species	Origin of Strains	NCBI
*Acinetobacter baumannii 11510*	Bronchial clinical isolate, Italy	NZ_CP018861.2
*Aerococcus urinae ACS-120-V-Col10a*	Human microbiota, USA	NC_015278.1
*Brevibacillus brevis DZQ7*	Tobacco rhizosphere soil, China	NZ_CP030117.1
*Brucella abortus 21630*	Buffalo lymph nodes, Italy	NZ_CP023235.1
*Chlamydia abortus 1H*	Clinical isolate, UK	NZ_LN554883.1
*Citrobacter freundii R17*	Wastewater treatment plant, China	NZ_CP035276.1
*Clostridium botulinum Mfbjulcb6*	Fish market isolate, India	CP027778.1
*Corynebacterium pseudotuberculosis Cap1R*	Goat intestine, Brazil	NZ_CP036258.1
*Edwardsiella ictaluri MS-17-156*	Catfish isolate, USA	NZ_CP028813.1
*Escherichia coli PPECC42*	Pig lung, China	NZ_CM003707.1
*Glutamicibacter nicotianae OTC-16*	Mud isolate, China	NZ_CP033081.1
*Haemophilus influenzae 5P54H1*	Clinical isolate, USA	NZ_CP020009.1
*Helicobacter pylori F209*	Human stomach, Japan	NZ_AP017332.1
*Klebsiella pneumoniae 121*	Human blood, China	NZ_CP031849.1
*Lactobacillus amylovorus GRL 1112*	Pig feces, Finland	NC_014724.1
*Lactobacillus reuteri ATG-F4*	Human isolate, South Korea	NZ_CP035790.1
*Listeria grayi NCTC 10812*	Corn leaves and stems, USA	NZ_LR134483.1
*Micrococcus luteus SB1254*	Marine isolate, South Korea	NZ_CP026366.1
*Mycobacterium abscessus FLAC013*	Human isolate, USA	NZ_CP014955.1
*Mycobacterium leprae TN*	Clinical isolate, USA	NC_002677.1
*Pandoraea pnomenusa MCB032*	Bioreactor isolate, UK	NZ_CP015371.1
*Propionibacterium acnes 6609*	Human isolate, Germany	NC_017535.1
*Pseudomonas aeruginosa Y71*	Clinical isolate, South Korea	NZ_CP030911.1
*Pseudomonas putida NX-1*	Soil isolate, China	NZ_CP030750.1
*Rickettsia typhi str. TH1527*	Clinical isolate, USA	NC_017066.1
*Salmonella enterica SA20100345*	Food isolate, Canada	NZ_CP022504.1
*Staphylococcus haemolyticus SGAir0252*	Air isolate, Singapore	NZ_CP025031.1
*Streptococcus pneumoniae NT_110_58*	Clinical isolate, Switzerland	NZ_CP007593.1
*Streptococcus pyogenes D471*	Human isolate, USA	NZ_CP011415.1
*Vibrio cholerae C5*	Clinical isolate, Indonesia	NZ_CP013301.1

## Data Availability

The original contributions presented in this study are included in the article/Supplementary Material. Further inquiries can be directed to the corresponding author.

## Supplementary Materials

The following supporting information can be downloaded at: https://www.mdpi.com/article/10.3390/microorganisms13061321/s1, Table S1: Overview of CRISPR-CAS systems in Archaea; Table S2: Overview of CRISPR-CAS systems in Bacteria; Table S3: Thermodynamics of direct repeats (DR) in Archaea; Table S4: Thermodynamics of direct repeats (DR) in Bacteria; Table S5: Plasmid sequences present in bacterial species lacking CRISPR-CAS systems; Table S6: Plasmid sequences present in bacterial species with confirmed CRISPR-CAS systems; Table S7: Plasmid sequences present in archaeal species with confirmed CRISPR-CAS systems; Figure S1: Phylogenetic tree of CAS1 proteins in archaeal and bacterial genomes; Figure S2: Representative RNA secondary structures of DRs in Archaea; Figure S3: Representative secondary structures of RNA from direct repeats (DR) in Bacteria; Figure S4: Secondary RNA structures of complete CRISPR arrays (DR + spacer) in Archaea; Figure S5: Secondary RNA structures of complete CRISPR arrays (DR + spacer) in Bacteria; Figure S6: Antibiotic resistance genes in archaeal genomes with confirmed CRISPR-CAS systems; Figure S7: Antibiotic resistance genes in bacterial genomes with confirmed CRISPR-CAS systems; Figure S8: Antibiotic resistance genes in bacterial species genomes lacking CRISPR-CAS systems.
