# Supplementary material for: Distribution of Genetic Determinants Associated with CRISPR-Cas Systems and Resistance to Antibiotics in the Genomes of Archaea and Bacteria
Source: Microorganisms. 2025 Jun 6;13(6):1321. doi: 10.3390/microorganisms13061321 (PMC12195208; doi:10.3390/microorganisms13061321)
Supplement: Supplementary file 1 [file microorganisms-13-01321-s001.zip › microorganisms-3677558-supplementary.pdf]

Table S1. Overview of CRISPR-CAS systems in Archaea

Species	Access number	CRISPR number	loci CRISPR: CRISPR length (pb)	Start	End	Spacers number	Spacers length (pb)	DRs number	DRs length (pb)	DR Consensus	Conserv. DR (%)	CRISPR Type	*cas* genes number	Origin of strain	Ref
Af. DSM_4304	NC_000917.1	3	4065	148	4213	59	35-44	60	30	GTTGAAATCAGACCAAAATGGGATTGAAAG	100	III-B / I-A	7 y 9	Sedimento hidrotermal.	Klenk y col., 1997
			3221	398369	401590	47	35-44	48	30	CTTTCAATCCCATTTTGGTCTGATTTCAAC	99				
			3251	1690930	1694181	43	35-41	10	37	GTAAGAAAGGGAGGCTCCTGAAAATGGAGATTGAAAG	98				
Fp. DSM_10642	NC_013849.1	6	603	273660	274263	9	40-45	10	23	CTTTCAATCCTCTTTTCATCGAG	98	I-A	5	Sedimento hidrotermal. Italia	Anderson y col., 2011
			1677	676127	677804	26	38-45	27	23	CTTTCAATCCTCTTTTCATCGAG	100				
			1138	824286	825424	17	39-50	18	24	GTTTCAGTCCTCTATTTGAGATTC	100				
			1362	1318858	1320220	21	38-43	22	23	CTCGATGAAAAGAGGATTGAAAG	100				
			655	1578276	1578931	10	39-42	11	23	CTTTCAATCCTCTTTTCATCGAG	98				
			1171	1597286	1598457	18	39-44	19	23	CTTTCAATCCTCTTTTCATCGAG	98				
Ga. SBH6	NZ_CP009552.1	3	973	1354028	1355001	14	36-41	15	30	CTTTCAATCCCTTAAAGGTCTGATTTCAAC	98	I-B	8	Sedimento hidrotermal. OA	Slobodkina y col., 2009
			843	1415496	1416339	12	36-40	13	30	CTTTCAATCCCTTAAAGGTCTGATTTTAAC	94				
			304	1644186	1644490	4	34-38	5	32	CTTTCAATCCCTTCAAGGTCTGATTTTAACGA	94				
Cd. PM4	NZ_LT719092.1	1	6141	1529346	1535487	92	27-38	93	30	ATTGTTAGAATACCTATAAGGACTTGAAAC	99	I-B	8	Sustratos ácidos. Europa	Golyshina y col., 2016
Fa. Fer1	NC_021592.1	2	904	15	919	13	36-39	14	30	ATTTCAATTCCTATATGGAATTATTTTAAC	100	I-B / I-B	9 y 7	Mina Richmond. USA	Allen y col., 2007
			498	1194568	1195066	7	35-39	8	30	GTGTTTAGTCTATCTATAAGGGTTTGAAAT	100				
Pt. DSM9790	NC_005877.1	4	294	15	309	4	36-37	5	28	CTTCCATACTATCTAGTAATTCTTAAAC	100	I-D / III-A	7 y 8	Suelo volcánico. Japón	Fütterery col., 2004
			990	51347	52337	14	37-47	15	28	CTTCCATACTATCTAGTAATTCTTAAAC	98				
			1142	61415	62557	16	38-48	17	30	CTTTCAATCCTATTTAGGTTATTATTTAAC	99				
			5555	1540318	1545873	82	35-44	83	28	CTTCCATACTAACTAGTACATCTTAAAC	99				
Tv. GSS1	NC_002689.2	2	1251	109188	110439	18	35-46	19	28	CTTCCATACTAACTAGTACATCTTAAAC	99	III-A	8	Sedimento hidrotermal. Italia	Kawashima y col., 2000
			1146	121470	122616	16	38-45	17	28	CTTCCATACTAACTAGTACATCTTAAAC	93				
Ms. ATCC35061	NC_009515.1	1	2887	152502	155389	43	32-38	44	31	ATTTCAATCCTATTATAGTCTTATTTTTAAC	100	I-B	8	Intestino humano	Samuel y col., 2007
Mst. DSM_3091	NC_007681.1	3	4057	490169	494226	61	36-41	62	29	GTTTAAAATAGACTTAATAGTATGAAAAC	97	I-B / I-B	8 y 7	Intestino humano	Fricke y col., 2006
			3753	1091284	1095037	56	30-43	57	30	AATAGAATAAGATCATAATGAAATTGAAAT	98				
			287	1095882	1096169	4	29-37	5	30	AATAGAATAAGATCATAATGAAATTGAAAT	76				
Mf. AG86	NC_013156.1	7	1446	281438	282884	21	34-42	22	30	CTTTCCATTCCGTTCCGGTCTGATTTTAAC	100	III-C / III-A /I-B / I-A	6, 5, 9 y 3	Sedimento hidrotermal. USA	Jeanthon y col., 1999
			501	347389	347890	7	34-43	8	30	GTTAAAATCAGACCGGAACGGTATGGAAAG	87				
			369	531678	532047	5	34-41	6	31	AATTTCCATTCCGAAACGGTCTGATTTTAAC	93				
			1108	675146	676254	16	33-42	17	30	ATTTCCATACCGTTCCGGTCTGATTTTAAC	99				
			962	717618	718580	14	34-39	15	30	GTTAAAATCAGACCGGAACGGTATGGAAAT	88				
			558	760023	760581	8	34-37	9	30	GTTAAAATAAGACCGTCTCGGTATCTAATA	93				
			298	1343405	1343703	4	35-39	5	30	GTTAAAATCAGACCGTCTCGGTATGGAAAT	100				

Cont.

Mi. Kol5	NC_015562.1	5	699	7992	8691	9	35-38	10	37	GTTTCCATCCTTGTTTTAATGGATGAGTAATTCAAAC	91	I-A / III-A / III-D	6, 6 y 5	Sedimento hidrotermal. USA	Burggraf y col., 1990
			713	220885	221598	10	35-40	11	31	GTTTCCATCCCCCTATGGGTCTGATTTTAAT	94				
			335	295799	296134	4	35-42	5	37	GTTTCCATCCTTGTTTTAATGGATGAGATATTCAAAC	92				
			1204	592560	593764	16	35-73	17	31	GTTAAAATCAGACCCTTAGGGGGATGGAAAT	92				
			1002	717057	718059	13	35-42	14	37	GTTTCCATCCTTGTTTTAATGGATGAGGTATTCAAAC	98				
Hli. tADL	NZ_CP024845.1	5	274	15756	16030	5	19-28	6	26	ACCTGAAGCACCTGCAGAGCCCGAAG	32	I-B	8	Antártida	Mou y col., 2012
			5671	1305778	1311449	86	33-38	87	30	GCTTCAACCTCACGAGAGTTCGTCTGAAAC	100				
			291	1320912	1321203	4	34-37	5	30	GCTTCAACCGCACAAGCGTTCGTCTGAAAC	83				
			1134	1474449	1475583	17	33-37	18	30	GTTTCAGACCAACCCTCGTGGGGTCGGAGG	96				
			1598	2597028	2598626	24	33-40	25	30	GTTTCAGACGAACTCTCGTGAGGTTGAAGC	100				
Ma. MRE50	NC_009464.1	1	6992	1749557	1756549	114	31-34	115	29	CGGTTCACCCCCACGCCTGTGGGGACTTT	98	I-E	7	Rizosfera de arroz	Erkel y col., 2006
Mb. MS2	NC_018227.2	1	10572	2627396	2637968	144	34-41	145	36	CTTACCATGTCTGAAAAGACATGGCTCCATTGAAGC	99	I-U	5	Lodo	Maus y col., 2012
Mp. E1-9c	NC_011832.1	2	211	1642478	1642689	3	31-32	4	29	CGGTTCATCCCCACGCTTGTGGGGAACTC	69	I-D / I-E	4 y 8	Pantano minerotrófico	Cadillo y col., 2015
			6192	1642843	1649035	101	31-32	102	29	CGGTTCATCCCCACGCTTGTGGGGAACTC	98				
Mh. JF-1	NC_007796.1	6	2150	841173	843323	30	33-38	31	36	GTTGCCATACCCTTCTATTTTCGGGTCACTTGCAAC	100	III-D / I-E / I-D	9, 9 y 9	Lodo	Gunsalus y col., 2016
			525	844463	844988	7	32-36	8	36	GTTGCCATACCCTTCTATTTTCGGGTCACTTGCAAC	100				
			2636	852826	855462	37	34-35	38	36	GTTGCCATACCCTTCTATTTTCGGGTCACTTGCAAC	96				
			4849	1568546	1573395	78	32	79	29	CGGTTCATCCCCATACACACGGGGAACTC	99				
			3161	2088864	2092025	43	35-37	44	37	GTTTCAATCCCTATCGGGTTTTCTTTTCCATTGTGAC	97				
			4762	2093152	2097914	65	34-38	66	37	GTTTCAATCCCTATCGGGTTTTCTTTTCCATTGTGAC	100				
Mac. C2A	NC_003552.1	5	473	2369218	2369691	6	35-32	7	37	ATTCGAGAGCAAGATCCACTAAAACAAGGATTGAAAC	100	III-A / I-B	5 y 7	Sedimento marino.USA	Galagan y col., 2002
			475	2378684	2379159	6	36-37	7	37	ATTCGCGAGCAAGATCCACTAAAACAAGGATTGAAAC	84				
			1937	2379328	2381265	26	30-41	27	37	ATTCGCGAGCAAGATCCACTAAAACAAGGATTGAAAC	92				
			108	4508268	4508376	1	35	2	37	GTTTCAATCCTTGTTTTAATGGATCTTGCTCTCGAAT	100				
			2029	4523522	4525551	30	34-41	31	30	GTTTCAATCCCTCTAAGGTCTGATTTTAAC	99				
Ne. SR1	NZ_CP007174.1	1	7220	2110971	2118191	99	31-52	100	37	GTTTCAATCCTTGTTGTGGTGGATTCGTCTTTCAGAC	100	I-B	6	Aguas termales	Zhalnina y col., 2014
Nca. SCU2	NZ_LT981265.1	3	6469	925831	932300	96	36-41	97	30	CTTTCAATCCTACTATAGTTCGATTCAAAC	100	I-B	7	Aguas termales. Italia	Abby y col., 2018
			451	1093286	1093737	4	58	5	44	GTTTGAGGAGGCTATAGCATGCTTTGATAAGGCTATAGA(	71				
			6816	1548627	1555443	101	36-40	102	30	ATTTCAATCCTACTATAGTTCGATTCAAAC	99				
Ap. K1	NC_000854.2	3	1749	717248	718997	26	37-52	27	24	GAATCTTCGAGATAGAATTGCAAG	96	I-A	7	Aguas termales	Kawarabayasi y col., 1999
			2698	786657	789355	41	36-47	41	25	GCATATCCCTAAAGGGAATAGAAAG	100				
			1187	1277299	1278486	18	38-45	19	24	CTTGCAATTCTATCTCGAAGATTC	97				
Ih. KIN4/I	NC_009776.1	10	357	89012	89369	5	36-46	6	26	CTTTCTATCCTCTCTTGAGACTCCAC	92	III-B / IIIB / I-A	3, 3 y 8	Aguas termales. Islandia	Podar y col., 2008
			628	260379	261007	9	37-41	10	26	CTTTCTATCCTCTTTTGAGACTCCAC	88				
			355	290621	290976	5	40-49	6	23	GAGTCTCAAAAGAGGATAGAAAG	92				
			160	388733	388893	2	41-42	3	26	CTTTCTATCCTCTTTTGAGACTCCAC	88				
			750	413553	414303	11	35-44	12	26	CTTTCTATCCTCTTTTGAGACTCCAC	80				
			563	557086	557649	8	36-46	9	26	GTGGAGTCTCAAAAGAGGATAGAAAG	83				
			421	915471	915892	6	40-46	7	23	GAGTCTCAAAAGAGGATAGAAAG	85				
			435	952490	952925	6	37-46	7	26	GTGGAGTCTCAAGAGAGGATAGAAAG	73				
			1455	955649	957104	23	36-42	24	24	GATTAAACGAAAGAGAACTGCAAG	92				
			428	1009879	1010307	6	37-44	7	26	CTTTCTATCCTCTTTTGAGACTTCAC	93				

Cont.

Ia. DSM 17230	NC_014471.1	7	576	536388	536964	8	43-47	9	24	CTTTCAATTCCATATATTGGATTC	100	I-A	9	Aguas termales. Nueva Zelanda	Niederberger y col., 2006
			656	546658	547314	9	44-50	10	24	GAATCCTATAAATGGAATTGAAAG	98				
			1328	562711	564039	19	41-51	20	24	CTTTCAATTCCATATATTGGATTC	96				
			1615	856468	858083	22	42-55	23	25	CTTTCTACTCCCTTTTGGGAGTTTC	94				
			1678	869202	870880	23	39-53	24	25	GAAACTCCCAAAAGGGAGTAGAAAG	100				
			2301	1242963	1245264	32	40-54	33	25	GAAACTCCCAAAAGGGAGTAGAAAG	100				
			936	1494064	1495000	13	42-51	14	24	GAATCCAATAAATGGAATTGAAAG	98				
Hb. DSM 5456	NC_008818.1	2	3306	632553	635859	48	37-55	49	25	GAACAACTCAAAAGAGAATTGCAAG	96	I-A / III-B	8 y 5	Aguas termales. Portugal	Brügger y col., 2007
			3182	702374	705556	46	39-53	47	25	CTTGCAATTCTCTTTTGAGTTGTTC	99				
Pd. Su06	NZ_CP013011.1	5	3574	286178	289752	53	36-49	54	25	GCATAACTCAAAAGAGAATTGTAAG	97	I-A	7	Chimenea hidrotermal. OP	Lin y col., 2018
			3515	307847	311362	51	39-47	52	25	CTTACAATTCTCTTTTGAGTTATGC	95				
			510	1335287	1335797	7	41-52	8	25	CTTTCAGTTCTATTTGTTATGATTC	93				
			573	1340092	1340665	8	41-47	9	25	GAATCATAACAAATAGAACTGAAAG	98				
			846	1358985	1359831	12	38-48	13	25	GAATCATAACAAATAGAACTGAAA	97				
Pf. 1A	NC_015931.1	7	2444	388807	391251	35	37-57	36	25	GAAACAACCAAGAATGAATTGAAAG	100	I-A / IIIB / I-A	4, 3 y 8	Respiradero hidrotermal	Anderson y col., 2011
			4719	425698	430417	69	37-52	70	25	CTTTCAATTCATTCTTTGTTGTTTC	97				
			1258	437565	438823	18	41-47	19	25	GAAACAACCAAGAATGAATTGAAAG	98				
			5457	461092	466549	79	39-58	80	25	CTTTCAATTCATTCTTGGTTGTTTC	100				
			2150	473827	475977	31	40-50	32	25	GAAACAACCAAGAATGAATTGAAAG	100				
			244	492539	492783	3	45-53	4	25	CTTTCAATTCATTCTTGGTTGTTTC	90				
			1185	501055	502240	17	39-53	18	25	CTTTCAATTCATTCTTGGTTGTTTC	100				
Ss. SULA	NZ_CP011057.1	4	7393	2112739	2120132	116	36-44	117	25	GATTAATCCCAAAAGGAATTGAAAG	100	I-A	13	Volcán Solfatara. Nápoles	McCarthy y col., 2015
			4462	2134655	2139117	70	34-44	71	25	CTTTCAATTCCTTTTGGGATTAATC	96				
			7980	2150493	2158473	126	37-44	127	24	GATAATCTCTTATAGAATTGAAAG	99				
			412	2557814	2558226	6	39-44	7	24	GATAATCTACTATAGAATTGAAAG	100				
Sa. HS-1	NZ_AP018553.1	2	14800	1664810	1679610	221	35-44	222	30	GTTTCAAGCCCTCAAAGGTAAGCTACAAAC	100	I-B / III-D / I-A	7, 7 y 6	Fuente termal ácida. Japón	Sakai y col., 2019
			1150	1679712	1680862	17	35-38	18	30	GTTTCAAGCCCTCAAAGGTAAGCTACAAAC	100				
Si. L.S.2.15	NC_012589.1	3	283	614481	614764	4	38-42	5	25	GATAAATCCCCAAAGGGATTGAGAG	88	III-B / III-D / I-A / III-B	6, 7, 13 y 7	Fuente termal ácida.	Keeling y col., 1996
			7878	619238	627116	122	36-44	123	24	CTTTCAATTCTATAGTAGATTAAC	99				
			6621	631174	637795	102	39-48	103	24	GTTAATCTACTATAGAATTGAAAG	99				
Tt. Kra1	NC_016070.1	7	1629	225270	226899	24	39-50	25	24	CCTTCAAATCTCTATCTGAGATTC	100	III-D / I-A	3 y 7	Fuente termal ácida.	Siebers y col., 2011
			434	317074	317508	6	41-45	7	26	AGTGGAAATCAAAAGATAGTAGAAAC	91				
			508	345344	345852	7	43-48	8	25	CTTTCTACTATCTTTTGATTTCCAC	84				
			2234	1075974	1078208	33	37-57	34	24	CTTTCAATCCTCTCTTTGAGATTC	99				
			2114	1081877	1083991	31	38-51	32	24	GAATCTCAAAGAGAGGATTGAAAG	100				
			1757	1095240	1096997	26	37-51	27	24	CTTTCAATCCTCTTTTTGAGATTC	94				
			1048	1103070	1104118	15	39-55	16	24	GAATCTCAAAGAGAGGATTGAAAG	100				
Vm. 768-28	NC_015151.1	4	2143	1377767	1379910	30	38-53	31	25	GATATTCTCTAAAGAGAATAGAAGT	93	I-A / III-B / III-A	8, 7 y 4	Fuente termal. Rusia	Gumerov y col., 2011
			861	1452274	1453135	12	43-51	13	25	CTTTCAATATTCTATTGAAATCAAC	98				
			1151	1469719	1470870	16	42-49	17	25	CTTTCAATATTCTATTGAAATCAAC	86				
			290	1482074	1482364	4	40-46	5	24	TTTCAATATTCTTTTGAAATCAAC	88				

The abbreviations *Af, Fp, Ga, Cd, Fa, Pt, Tv, Ms, Mst, Mf, Mi, Hli, Ma, Mb, Mp, Mh, Mac, Ne, Nca, Ap, Ih, Ia, Hb, Pd, Pf, Ss, Sa, Si, Tt* and *Vm* correspond to the species *Archaeoglobus fulgidus, Ferroglobus placidus, Geoglobus acetivorans, Cuniculiplasma divulgatum, Ferroplasma acidarmanus, Pycrophilus torridus, Thermoplasma volcanium, Methanobrevibacter smithii, Methanosphaera stadtmanae, Methanocaldococcus fervens, Methanotorris igneus, Halohasta litchfieldiae, Methanocella arvoryzae, Methanoculleus bourgensis, Methanosphaerula palustris, Methanospirillum hungatei, Methanosarcina acetivorans, Nitrososphaera evergladensis, Nitrosocaldus cavascurensis, Aeropyrum pernix, Ignicoccus hospitalis, Ignisphaera aggregans, Hyperthermus butylicus, Pyrodictium delaneyi, Pyrolobus fumarii, Saccharolobus solfataricus, Sulfodiicococcus acidiphilus, Sulfolobus islandicus, Thermoproteus tena*x and *Vulcanisaeta moutnovskia*, respectively.

Table S2. Overview of CRISPR-CAS systems in Bacteria

			loci CRISPR												
Species	Access number	CRISPR number	CRISPR length (pb)	Start	End	Spacers number	Spacers length (pb)	DRs number	DRs length (pb)	DR Consensus	Conserv. DR (%)	CRISPR Type	*cas* genes number	Origin of strain	Ref
Ab. A388	NZ_CP024418.1	1	3209	1173172	1176381	53	32	54	28	GTTCATGGCGGCATACGCCATTTAGAAA	100	I-F	6	Aislado clínico.	Fournier y col., 2006
Pa. UCBPP-PA14	NC_008463.1	2	873	2926520	2927393	14	32-38	15	28	GTTCACTGCCGTATAGGCAGCTAAGAAA	86	I-F	6	Aislado clínico. Massachusetts, USA.	Lee y col., 2006
			1288	2935917	2937205	21	32-33	22	28	TTTCTTAGCTGCCTACACGGCAGTGAAC	100				
Ec. K-12	NZ_LN832404.1	2	762	2874005	2874767	12	32-33	13	29	CGGTTTATCCCCGCTGGCGCGGGGAACTC	92	I-E	8	Intestino humano	Gilson y col., 1982
			393	2900318	2900711	6	33	7	28	GGTTTATCCCCGCTGGCGCGGGGAACAC	98				
Pm. FDAARGOS_67	NZ_CP026051.1	2	333	946311	946644	5	32	6	29	CGGTTCATCCCCGTATACACGGGGAACAC	91	I-E	8	Intestino humano	Di Pilato y col., 2016
			517	955532	956049	8	32	9	29	CGGTTCATCCCCGTGCATACGGGGAACAC	97				
Sm. N4-5	NZ_CP031316.1	2	507	442146	442653	8	32	9	28	GTGCACTGCCGTACAGGCAGCTTAGAAA	91	I-F	6	Aislado clínico	Matilla y col., 2017
			1047	451133	452180	17	32	18	28	GTTCACTGCCGCATAGGCAGCTTAGAAA	90				
Ecl. C2-1	:NZ_CP035738.1	2	1049	3268882	3269931	17	32-33	18	28	TTTCTAAGCTGCCTGTACGGCAGTGCAC	93	I-F	6	Aislado clínico	Shankar y col., 2012
			628	3279384	3280012	10	32	11	28	TTTCTAAGCTGCCTGTACGGCAGTGAAC	89				
Kp. AATZP	NZ_CP014755.1	2	2649	4233882	4236531	43	29-32	44	29	CGGTTTATCCCCGCTGGCGCGGGGAACAC	96	I-E	8	Aislado clínico	Conlan y col., 2016
Ef. SRCM103470	NZ_CP035222.1	1	497	299515	300012	7	30	8	36	GTTTTAGAGCTATGCTGATTTGAATGCTTCCAAAAC	89	II-A	4	Aislado clínico	Lam y col., 2012
Efa. OG1RF	NC_017316.1	2	498	422484	422982	7	29	8	37	GTTTTAGAGTCATGTTGTTTAGAATGGTACCAAAACT	85	II-A	4	Aislado clínico	Bourgogne y col., 2008
			498	1738161	1738659	7	29	8	37	GGTTTTGGTACCATTCTAAACAACATGACTCTAAAAC	80				
Se. FDAARGOS_153	NZ_CP014119.1	2	324	959268	959592	4	34-36	5	37	TGTTCTCGTCCCCTTTTCGGCGGGGTGGTTAGCGAAT	81	III-A	9	Aislado clínico	Goldberg y col., 2017
			822	968313	969135	11	34-37	12	36	GTTCTCGTCCCCTTTTCTTCGGGGTGGTTATCGATC	90				
Hm. NCTC12198	NC_013949.1	1	694	24706	25400	10	29-30	11	36	GTTTTAGCCACTTCATAAATATGTTTATGCTAAAAT	100	II-C	3	Aislado de Turón europeo.	O'Toole y col., 2010
Cj. NS4-5-1	NZ_CP007192.1	1	366	1458065	1458431	5	30-31	6	36	GTTTTAGTCCCTTTTTAAATTTCTTTATGGTAAAAT	98	II-C	3	ND	Timms y col., 2014
Sen. SA19980677	NZ_CP007285.2	2	638	3082388	3083026	10	32	11	29	CGGTTTATCCCCGCTGGCGCGGGGAACAC	94	I-E	8	Aislado ambiental. Canadá	Rehman y col., 2014
			1614	3099158	3100772	26	32	27	29	CGGTTTATCCCCGCTGGCGCGGGGAACAC	91				
Nm. NCTC10025	NZ_LR134525.1	1	1223	1523634	1524857	18	30	19	36	ATTGTAGCACTGCGAAATGAGAAAGGGAGCTACAAC	98	II-C	3	Fluido cerebroespinal humano.	Branham y col., 1967
Hi. NCTC11873	NZ_LR134490.1	1	530	1063578	1064108	7	31-36	8	37	GTCGAAAGACATTGCCCTGTTCCAAAGGGATTGAGAC	99	III-A	8	Aislado clínico	Fleischmann y col., 1995
Sp. MGAS23530	CP013839.1	1	365	756938	757303	5	30	6	36	GTTTTAGAGCTATGCTGTTTTGAATGGTCCCAAAAC	91	II-A / I-C	4 y 7	Aislado clínico	Smoot y col., 2002
Sd. CFSAN010956	NZ_CP026827.1	1	90	198889	198979	1	31	2	30	TGTGTTCCCCGCGCCAGCGGGGATAAACCG	100	I-E	8	Aislado clínico	Khan y col., 2014
Na. NCTC12227	NZ_LR134516.1	1	431	1231419	1231850	6	30	7	36	ATTGTAGCACTACGAGATGAGAGAGGAAGCTACAAC	100	II-C / I-C	3 y 5	Aislado clínico animal	Vandamme y col., 2006
Vv. YJ016	NC_005140.1	2	175	1691478	1691653	2	34-37	3	35	GTTTCAGACATGCCCGGTTTAGACGGGATTAAGAC	92	III-D	7	Aislado clínico. Taiwán	Chen y col., 2003
			661	1694586	1695247	9	33-37	10	35	GTTTCAGACATGCCCGGTTTAGACGGGATTAAGAC	100				

Cont.

Vp. FORC_022	NZ_CP013249.1	1	1707	826587	828294	28	32	29	28	GTTAACTGCCACACAGGCAGCTTAGAAA	98	I-F	6	Aislado de cangrejos marinos.	Lee y col., 2015
Vc. FORC_076	NZ_CP026531.1	1	4347	830677	835024	72	32	73	28	GTTCACTGCCGCACAGGCAGCTTAGAAA	96	I-F	6	Aislado acuático	Chung y col., 2018
Bc. KSM-K16	NC_006582.1	6	760	3668511	3669271	11	33-35	12	32	ATTTCAATCCACGCACTCACAAAGAGTGCGAC	95	I-C	7	Aislado de suelo.	Takaki y col., 2003
			1028	3720845	3721873	15	33-35	16	32	ATTTCAATCCACGCACTCACAAAGAGTGCGAC	99				
			696	3728825	3729521	10	34-37	11	32	ATTTCAATCCACGCACTCACATAGAGTGCGAC	93				
			1164	3739183	3740347	17	33-36	18	32	ATTTCAATCCACGCACTCATATAGAGTGCGAC	96				
			563	3741040	3741603	8	34-35	9	32	ATTTCAATCCACGCACTCATATAGAGTGCGAC	83				
			425	3820899	3821324	6	33-35	7	32	ATTTCAATCCACGCACTCACTAAGAGTGCGAC	85				
La. YT1	NZ_CP025200.1	2	1678	546310	547988	27	32	28	29	GTTTTCTCCACGTATGTGGAGGTGATCCT	93	I-E	7	Aislado ambiental	Nam y col., 2017
			1678	557572	558392	13	33	14	28	ATTTTCTCCACGTATGTGGAGGTGATCC	79				
Li. Clip11262	NC_003212.1	1	695	2768992	2769687	10	30	11	36	GTTTTGTTAGCATTCAAAATAACATAGCTCTAAAAC	91	II-A	2	Productos lácteos. Marruecos	Glaser y col., 2001
Lm. Lm3163	NZ_CP013722.1	3	2886	976133	979019	44	35-37	45	29	ATTTACATTTCATAATAAGTAGTTAAAAC	98	I-B / II-A	8 y 4	Aislado clínico. Suiza	Tasara y col., 2015
			476	996714	997190	7	36-37	8	28	ATTTACATTTCACAATAAGTAACTAAAA	100				
			1422	1793158	1794580	21	30	22	36	GTTTTAGAGCTATGTTATTTTGAATGCTACCAAAAC	93				
Mp. 1049	NZ_CP033058.2	2	1095	449344	450439	16	30-31	17	36	GTTTTAGTACTATACTATTTTTAAGCAATACAAAAC	100	II-C	1	Cerebro de Focas	Frasca y col., 2018
			567	451968	452535	8	30-32	9	36	GTTTTAGTACTATACTATTTTTAAGCAATACAAAAC	83				
Af. DSM 20731	NC_013740.1	1	4019	882151	886170	61	33-37	62	31	GTCGCCCCCGCAAGGGGGCGTGGATTGAAAT	93	I-C	7	Intestino de cerdos	Chang y col., 2010
Bco. HM-08	NZ_CP010525.1	2	1420	709156	710576	21	33-35	22	32	GTCGCTCCCTACATGGGGGCGTGGATTGAAAT	100	I-C	7	Productos lácteos	Zhang y col., 2015
			1157	710677	711834	17	34-36	18	32	GTCGCTCCCTACATGGGGGCGTGGATTGAAAT	100				
Mc. BBH18	NC_014147.1	2	209	27785	27994	3	32	4	28	TTTCTAAGCGACCTGTGCGGTCGTGAAG	97	I-F	6	Aislado clínico	De Vries y col., 2010
			2911	28086	30997	48	32-33	49	28	TTTCTAAGCGACCTGTGCGGTCGTGAAG	99				
Yp. FDAARGOS_602	NZ_CP033696.1	1	268	11641	11909	4	32-33	5	28	TTTCTAAGCTGCCTGTGCGGCAGTGAAC	94	I-F	6	Aislado de pulgas.	Parkhill y col., 2001

The abbreviations *Ab, Pa, Ec, Pm, Sm, Ecl, Kp, Ef, Efa, Se, Hm, Cj, Sen, Nm, Hi, Sp, Sd, Na, Vv, Vp, Vc, Bc, La, Li, Lm, Mp, Af, Bco, Mc* and *Yp* correspond to the species *Acinetobacter baumanii, Pseudomonas aeruginosa, Escherichia coli, Proteus mirabilis, Serratia marcescens, Enterobacter cloacae, Klebsiella pneimoniae, Enterococcus faecium, Enterococcus faecalis, Staphylococcus epidermidis, Helicobacter mustelae, Campylobacter jejuni, Salmonella enterica, Neisseria meningitidis, Haemophilus influenzae, Streptococcus pyogenes, Shigella dysenterae, Neisseria amenaloris, Vibrio vulnificus, Vibrio parahaemolyticus, Vibrio cholerae, Bacillus claussi, Lactobacillus acidophilus, Listeria innocua, Listeria monocytogenes, Mycoplasma phocicerebrales, Acidaminococcus fermentans, Bacillus coagulans, Moraxella catarrhalis* and *Yersinia pestis*, respectively. ND: Not determined.

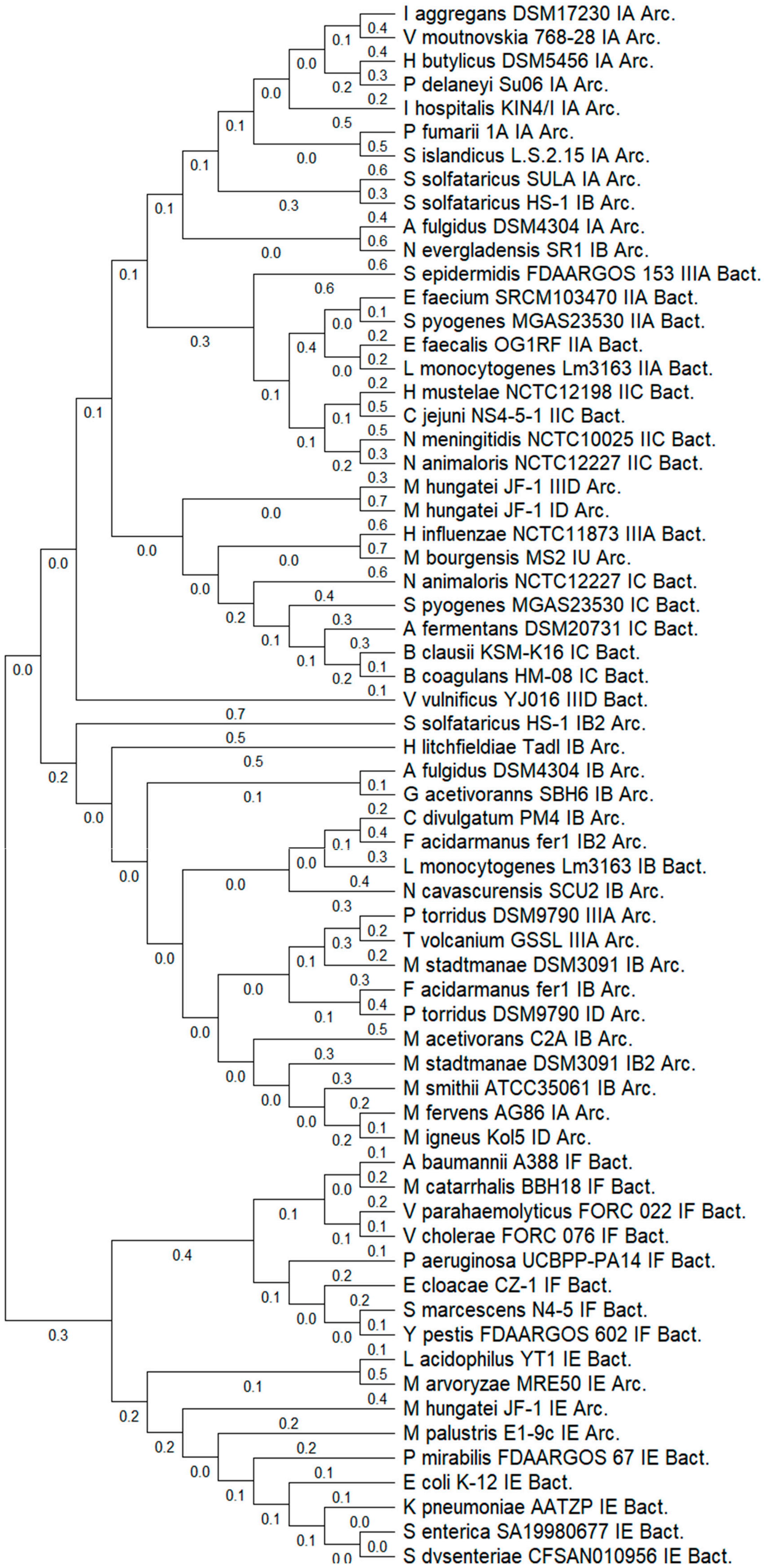

Figure S1. Phylogenetic tree of CAS1 proteins in archaeal and bacterial genomes. The UPGMA tree of the CAS1 protein was generated using the MUSCLE algorithm in MEGA12. Representative CAS1 proteins from all identified subtypes were selected. Evolutionary history was inferred using the Neighbor-Joining method. The optimal tree with the sum of branch length = 26.482 is shown (below the branches). The evolutionary distances were computed using the Poisson correction method and are in the units of the number of amino acid substitutions per site. The analytical procedure encompassed 65 amino acid sequences. The pairwise deletion option was applied to all ambiguous positions for each sequence pair resulting in a final data set comprising 787 positions.

Table S3. Thermodynamics of direct repeats (DR) in Archaea

Species	CRISPR	Sequence type	DR Sequence (5'-3')	ΔG (kcal/mol)	ΔH (kcal/mol)	ΔS cal/(K·mol)	T_m (°C)	MFE DR (kcal/mol)	DR folding kinetics (structures)	MFE CRISPR (kcal/mol)
Af. DSM_4304	1	Consensus	GTTGAAATCAGACCAAAATGGGATTGAAAG	-2.60	-54.50	-167.3	52.5	-3.82	924	-1181.74
	2	Consensus	CTTTCAATCCCATTTTGGTCTGATTTCAAC	-2.60	-54.50	-167.3	52.5	-2.82	714	-845.74
		Variant	CTTTTAATCCCATTTTGGTCTGATTTCAAC	-2.60	-54.50	-167.3	52.5	-1.80	847	
	3	Consensus	GTAAGAAAGGGAGGCTCCTGAAAATGGAGATTGAAAG	-5.10	-61.50	-181.8	65	-5.42	1895	-1065.64
		Variant	ATAAGAAAGGGAGGCTCCTGAAAATGGAGATTGAAAG	-5.10	-61.50	-181.8	65	-5.42	1833	
		Variant	ATTAAGCTGCCGTATTCCTGAAAATGGAGATTGAAAG	-4.00	-62.50	-188.6	58.2	-4.27	10843	
Fp. DSM_10642	1	Consensus	CTTTCAATCCTCTTTTCATCGAG	-0.70	-28.30	-88.9	44.8	-0.92	71	-200.04
		Variant	TTTTCAATCCTCTTTTCATCGAG	-0.70	-28.30	-88.9	44.8	-0.92	93	
	2	Consensus	CTTTCAATCCTCTTTTCATCGAG	-0.70	-28.30	-88.9	44.8	-0.92	71	-547.69
	3	Consensus	GTTTCAGTCCTCTATTTGAGATTC	-3.60	-46.80	-139.2	62.8	-3.83	214	-387.71
	4	Consensus	CTCGATGAAAAGAGGATTGAAAG	-2.40	-38.20	-115.4	57.7	-2.78	62	-430.53
	5	Consensus	CTTTCAATCCTCTTTTCATCGAG	-0.70	-28.30	-88.9	44.8	-0.92	71	-188.99
		Variant	CTTTCAATCCTCTTTTCATCAAG	1.40	-17.00	-59.3	13.4	-0.04	53	
	6	Consensus	CTTTCAATCCTCTTTTCATCGAG	-0.70	-28.30	-88.9	44.8	-0.92	71	-341.26
		Variant	ATTTCAATCCTCTTTTCATCGAG	-0.70	-28.30	-88.9	44.8	-0.92	81	
Ga. SBH6	1	Consensus	CTTTCAATCCCTTAAAGGTCTGATTTCAAC	-2.70	-55.80	-171.2	52.7	-2.93	683	-294.81
		Variant	CTTTCAATCCCTTCAAGGTCTGATTTCAAC	-2.70	-55.80	-171.2	52.7	-2.94	584	
	2	Consensus	CTTTCAATCCCTTAAAGGTCTGATTTTAAC	-2.70	-55.80	-171.2	52.7	-2.95	899	-251.21
		Variant	CTTTCGGTCTCTTAAAGGTTTGATTTTAAC	-2.30	-62.00	-192.4	48.9	-1.55	2206	
	3	Consensus	CTTTCAATCCCTTCAAGGTCTGATTTTAACGA	-2.70	-55.80	-171.2	52.7	-2.94	2001	-104.79
		Variant	CTTTCAATCCCTTCAAGGTCTGATTTTAACCT	-2.90	-50.60	-153.7	55.8	-3.00	762	
Cd. PM4	1	Consensus	ATTGTTAGAATACCTATAAGGACTTGAAAC	-2.10	-29.60	-88.6	60.6	-1.50	1218	-1351.34
		Variant	ATTGTTAGAATACCCATAAGGACTTGAAAA	-1.10	-27.20	-84.1	50	-1.18	1054	
Fa. Fer1	1	Consensus	ATTTCAATTCCTATATGGAATTATTTTAAC	-5.70	-58.70	-170.8	70.3	-5.44	1392	-237.03
	2	Consensus	GTGTTTAGTCTATCTATAAGGGTTTGAAAT	-3.00	-58.00	-177.3	53.9	-1.45	4291	-174.28
Pt. DSM9790	1	Consensus	CTTCCATACTATCTAGTAATTCTTAAAC	-2.00	-37.20	-113.4	54.6	-0.95	234	-83.90
	2	Consensus	CTTCCATACTATCTAGTAATTCTTAAAC	-2.00	-37.20	-113.4	54.6	-0.95	234	-225.60
		Variant	ACTCCATACTATCTAGTAATTCTTAAAC	-2.00	-37.20	-113.4	54.6	-0.95	125	
	3	Consensus	CTTTCAATCCTATTTAGGTTATTATTTAAC	-3.30	-34.70	-101.2	69.5	-1.46	967	-274.92
		Variant	CTTCCATACTATCTATTAATTCTTAAAC	0.10	-26.70	-86.4	35.8	-0.06	121	
	4	Consensus	CTTCCATACTAACTAGTACATCTTAAAC	-1.70	-33.20	-101.5	53.7	-0.81	91	-1111.40
		Variant	TTTTCAATCCTATTTAGGTTATTATTTAAC	-3.30	-34.70	-101.2	69.5	-1.46	1268	
Tv. GSS1	1	Consensus	CTTCCATACTAACTAGTACATCTTAAAC	-1.70	-33.20	-101.5	53.7	-0.81	91	-306.70
		Variant	TTTCCATACTAACTAGTACATCTTAAAC	-1.70	-33.20	-101.5	53.7	-0.81	164	
	2	Consensus	CTTCCATACTAACTAGTACATCTTAAAC	-1.70	-33.20	-101.5	53.7	-0.81	91	-274.05
		Variant	CTTCCACACCAATTAGTACGTCTCAAAC	0.60	-26.20	-86.4	30	-0.14	44	
Ms. ATCC35061	1	Consensus	ATTTCAATCCTATTATAGTCTTATTTTTAAC	0.40	-19.30	-63.5	30.7	-0.17	785	-477.47

Mst. **DSM_3091**	1	Consensus	GTTTAAAATAGACTTAATAGTATGAAAAC	-1.90	-33.00	-100.2	55.9	-1.27	1612	-779.07
		Variant	GTTTAAAATAGACTTAATA**A**TATGAAAAC	-1.90	-33.00	-100.2	55.9	-1.07	1274	
		Variant	GTTTAAAATAGACTTAAT**T**GTA**AAACCAA**	-2.50	-48.80	-149.2	53.7	-1.24	1241	
	2	Consensus	AATAGAATAAGATCATAATGAAATTGAAAT	-1.10	-29.20	-90.6	49.1	-0.58	616	-647.35
		Variant	AATAGAA**A**TA**GAATAAG**AT**C**A**T**A**A**TGAAAT	-1.90	-50.70	-157.3	49	-0.28	289	
	3	Consensus	AATAGAATAAGATCATAATGAAATTGAAAT	-1.10	-29.20	-90.6	49.1	-0.58	616	-81.40
		Variant	AATAGAATAAGATCAT**GTGA**AATTGAAAT**A**	-0.90	-32.50	-101.8	45.8	-0.93	615	
		Variant	AATAGAATAAGATCATAATGAA**T**TTGAAAT	-2.40	-44.40	-135.4	54.7	-0.71	1035	
		Variant	AATA**A**AATAAGA**AT**A**AG**A**TCATC**TTGAAAT	-2.60	-37.90	-113.8	59.8	-1.21	563	
Mf. **AG86**	1	Consensus	CTTTCCATTCCGTTCCGGTCTGATTTTAAC	-2.60	-47.10	-143.4	55.1	-3.17	311	-372.82
	2	Consensus	GTTAAAATCAGACCGGAACGGTATGGAAAG	-6.30	-52.60	-149.2	79.2	-4.88	380	-160.96
		Variant	GTTAAAATCAGACCGGAACGGTATGGAAA**T**	-6.30	-52.60	-149.2	79.2	-4.89	551	
		Variant	GTTAAAAT**A**AGAC**A**G**TCT**CGGTATGGAAA**C**	-2.30	-66.30	-206.3	48.1	-1.18	780	
	3	Consensus	AATTTCCATTCCGAAACGGTCTGATTTTAAC	-2.60	-47.10	-143.4	55.1	-3.24	1405	-105.38
		Variant	**TG**TTTCCATTCCGAAACGGTCTGATTTTAAC	-4.60	-48.50	-141.5	69.4	-4.27	1748	
		Variant	**C**ATTTCCATTCCGAAACGGTCTGATTTTAAC	-2.60	-47.10	-143.4	55.1	-3.18	831	
		Variant	**T**ATTTCCATTCCGAAACGGTCTGATTTTAAC	-2.60	-47.10	-143.4	55.1	-3.19	1049	
	4	Consensus	ATTTCCATACCGTTCCGGTCTGATTTTAAC	-2.60	-47.10	-143.4	55.1	-4.40	416	-263.12
		Variant	ATTTCCATACCGTTCCG**A**TCTGATTTTAAC	0.20	-18.90	-61.5	33.7	-0.20	283	
	5	Consensus	GTTAAAATCAGACCGGAACGGTATGGAAAT	-6.30	-52.60	-149.2	79.2	-4.89	551	-258.53
		Variant	GTTAAAATCAGACCGGAACGG**A**ATGGAAA**C**	-3.80	-51.00	-152.1	61.9	-3.52	319	
		Variant	GTTAAAATCAGA**GTCTT**A**GA**GAATG**A**AAAT	-4.00	-59.50	-178.9	59.3	-1.03	984	
	6	Consensus	GTTAAAATAAGACCGTCTCGGTATCTAATA	-4.00	-36.10	-103.4	75.6	-3.48	1200	-159.64
		Variant	GTTAAAATAAGACCGTCTCGGTAT**GG**AA**AC**	-4.00	-36.10	-103.4	75.6	-3.36	1057	
	7	Consensus	GTTAAAATCAGACCGTCTCGGTATGGAAAT	-6.30	-52.60	-149.2	79.2	-4.89	1146	-105.04
Mi. **Kol5**	1	Consensus	GTTTCCATCCTTGTTTTAATGGATGAGTAATTCAAAC	-5.20	-57.70	-169.2	67.7	-6.22	27454	-214.85
		Variant	GTTT**AT**ATCCTTGTTTTAATGGATGAGTAATTCAAAC	-3.80	-50.20	-149.6	62.4	-4.16	44857	
	2	Consensus	GTTTCCATCCCCCTATGGGTCTGATTTTAAT	-3.60	-37.80	-110.2	69.6	-4.44	1486	-214.91
		Variant	**A**TTT**T**CATCCCCCTATGGGTCTGATTTTAA**C**	-5.20	-66.80	-198.6	63.1	-5.27	1102	
		Variant	**A**TTTCCATCCCCCTATGGGTCTGATTTTAA**C**	-3.60	-37.80	-110.2	69.6	-4.43	867	
		Variant	GTTTCCATCCCCCTATGGGTCTGATTTTAA**C**	-3.60	-37.80	-110.2	69.6	-4.51	1289	
	3	Consensus	GTTTCCATCCTTGTTTTAATGGATGAGATATTCAAAC	-6.00	-82.20	-245.6	61.4	-7.66	38228	-114.25
		Variant	GTTTCCA**G**CC**A**TGTTTTAATGGAT**A**AG**G**TATTCAAAC	-4.90	-48.10	-139.2	72.1	-5.31	29111	
	4	Consensus	GTTAAAATCAGACCCTTAGGGGGATGGAAAT	-6.50	-65.20	-189.2	71.3	-8.17	2190	-393.40
		Variant	GTT**TC**AAT**T**AG**AG**CCTTAGGGG**AG**T**AA**AAAT	-2.30	-33.60	-100.9	59.7	-2.39	4447	
	5	Consensus	GTTTCCATCCTTGTTTTAATGGATGAGGTATTCAAAC	-6.00	-82.20	-245.6	61.4	-7.16	44052	-316.93
		Variant	GTTTCCATCCTTGTTTTAATGGATGAG**A**TATTCAAAC	-6.00	-82.20	-245.6	61.4	-7.66	38228	
Hli. **tADL**	1	Consensus	ACCTGAAGCACCTGCAGAGCCCGAAG	-2.30	-35.10	-105.7	58.7	-1.80	42	-184.86
		Variant	**T**CC**GC**A**G**GCACC**GA**C**G**GAGCCCGAAG	-5.30	-44.30	-125.7	79.1	-5.29	69	
		Variant	**G**CCTGAAGC**G**CC**AGCC**GA**A**CC**T**GAAG	-2.50	-56.60	-174.4	51.3	-2.27	54	
		Variant	**G**CC**C**GAAG**G**ACCTGCAGA**T**CC**T**GA**T**G	-3.50	-41.50	-122.5	65.5	-3.16	87	
		Variant	**G**CC**A**GAAGAACC**G**G**AC**GAG**T**CCGA**G**G	-4.40	-43.30	-125.4	72	-4.27	35	
	2	Consensus	GCTTCAACCTCACGAGAGTTCGTCTGAAAC	-3.50	-84.80	-262.1	50.3	-3.34	685	-1868.13
	3	Consensus	GCTTCAACCGCACAAGCGTTCGTCTGAAAC	-3.50	-84.80	-262.1	50.3	-4.39	511	-161.41
		Variant	**GAACGG**ACC**C**CACAAGCGTTCGTCTGAAAC	-5.20	-73.40	-219.8	60.6	-5.61	231	
		Variant	GCTTCAACCGCACAAGCGTTCGTCTGAAA**G**	-4.10	-76.30	-232.7	54.6	-4.44	846	
	4	Consensus	GTTTCAGACCAACCCTCGTGGGGTCGGAGG	-9.40	-69.30	-193.1	85.6	-9.01	3817	-514.89
		Variant	GTTTCAGACCAACCCTCGT**A**GGTCGGAG**TT**	-7.60	-69.60	-199.9	75	-7.74	1528	
	5	Consensus	GTTTCAGACGAACTCTCGTGAGGTTGAAGC	-6.90	-89.90	-267.6	62.7	-7.01	4553	-717.93

***Ma.* MRE50**	1	Consensus	CGGTTCACCCCCACGCCTGTGGGGACTTT	**-11.00**	**-74.10**	**-203.4**	**91**	**-11.72**	**1976**	**-2908.89**
		Variant	TTGTTCACCCCCACATCCGTGGGGACCTA	**-11.10**	**-74.20**	**-203.4**	**91.5**	**-11.62**	**886**	
		Variant	AGGTTCACCCCCACGCCTGTGGGGACTTT	**-11.00**	**-74.10**	**-203.4**	**91**	**-11.82**	**3855**	
		Variant	TGGTTCACCCCCACGCCTGTGGGGACTTT	**-11.00**	**-74.10**	**-203.4**	**91**	**-11.71**	**2159**	
***Mb.* MS2**	1	Consensus	CTTACCATGTCTGAAAAGACATGGCTCCATTGAAGC	**-13.30**	**-83.00**	**-224.7**	**96.1**	**-12.38**	**5172**	-
		Variant	CTTACCATGTCTTTTCAGACATGGCTCCATTGAAGC	**-10.10**	**-76.60**	**-214.4**	**84.1**	**-11.08**	**8514**	
***Mp.* E1-9c**	1	Consensus	CGGTTCATCCCCACGCTTGTGGGGAACTC	**-13.10**	**-105.30**	**-297.2**	**81**	**-13.36**	**2396**	**-126.54**
		Variant	CGGTTCATCCCTTCACTTGTGAGGAACCC	**-7.00**	**-93.80**	**-279.8**	**62**	**-6.17**	**1111**	
		Variant	CGGTTCATCCCCACGCTTGTGGGGAACAG	**-13.00**	**-85.40**	**-233.4**	**92.6**	**-13.05**	**1180**	
		Variant	CGGTTTATCCCCACAAGCGTGGGGAACTC	**-13.20**	**-105.40**	**-297.2**	**81.4**	**-13.34**	**2102**	
	2	Consensus	CGGTTCATCCCCACGCTTGTGGGGAACTC	**-13.10**	**-105.30**	**-297.2**	**81**	**-13.36**	**2396**	**-2792.65**
		Variant	TAGTTCATCCTCACGCTTGTGGGGAACTC	**-10.50**	**-79.00**	**-220.8**	**84.5**	**-10.34**	**1740**	
		Variant	AGGTTCATCCCCACGCTTGTGGGGAACTC	**-13.10**	**-105.30**	**-297.2**	**81**	**-13.41**	**2893**	
		Variant	TGGTTCATCCCCACGCTTGTGGGGAACTC	**-13.10**	**-105.30**	**-297.2**	**81**	**-13.32**	**2612**	
***Mh.* JF-1**	1	Consensus	GTTGCCATACCCTTCTATTTTCGGGTCACTTGCAAC	**-8.20**	**-92.00**	**-270.1**	**67.3**	**-8.19**	**2830**	**-662.97**
	2	Consensus	GTTGCCATACCCTTCTATTTTCGGGTCACTTGCAAC	**-8.20**	**-92.00**	**-270.1**	**67.3**	**-8.19**	**2830**	**-187.84**
	3	Consensus	GTTGCCATACCCTTCTATTTTCGGGTCACTTGCAAC	**-8.20**	**-92.00**	**-270.1**	**67.3**	**-8.19**	**2830**	**-844.52**
		Variant	GGTGGAATATACTTCTAATTTCGGGTCTCTTGCAAC	**-3.40**	**-73.50**	**-226**	**52**	**-2.39**	**11481**	
		Variant	GTTGCCATACCCTTCTATTTTAGGGTCACTTGCAAC	**-9.10**	**-93.60**	**-272.4**	**70.4**	**-10.80**	**6107**	
	4	Consensus	CGGTTCATCCCCATACACACGGGGAACTC	**-9.10**	**-84.70**	**-243.7**	**74.3**	**-9.17**	**621**	**-1690.77**
		Variant	TGGTTCATCCCCATACACACGGGGAACTC	**-9.10**	**-84.70**	**-243.7**	**74.3**	**-9.12**	**737**	
	5	Consensus	GTTTCAATCCCTATCGGGTTTTCTTTTCATTGTGAC	**-3.30**	**-34.80**	**-101.5**	**69.4**	**-4.35**	**6238**	**-962.61**
		Variant	GTTTCAATCCCTATCGGGTTTTCTTTTAGGAGTTACG	**-6.60**	**-78.20**	**-230.8**	**65.5**	**-5.62**	**26675**	
	6	Consensus	GTTTCAATCCCTATCGGGTTTTCTTTTCATTGTGAC	**-3.30**	**-34.80**	**-101.5**	**69.4**	**-4.35**	**6238**	**-1320.75**
***Mac.* C2A**	1	Consensus	ATTCGAGAGCAAGATCCACTAAAACAAGGATTGAAAC	**-4.70**	**-52.30**	**-153.4**	**67.6**	**-3.43**	**1455**	**-142.74**
	2	Consensus	ATTCGCGAGCAAGATCCACTAAAACAAGGATTGAAAC	**-4.70**	**-52.30**	**-153.4**	**67.6**	**-3.43**	**1455**	**-140.80**
		Variant	ATTCGCGAGCAAGATCCACTAAGAGCCTATCCGAAAA	**-3.90**	**-104.50**	**-324.3**	**49**	**-3.82**	**983**	
	3	Consensus	ATTCGCGAGCAAGATCCACTAAAACAAGGATTGAAAC	**-4.70**	**-52.30**	**-153.4**	**67.6**	**-3.43**	**1455**	**-506.72**
		Variant	GTTGCAAAACAGCTTCCAGCAAAATAAGGATTGAAAC	**-6.30**	**-64.40**	**-187.3**	**70.6**	**-3.47**	**4997**	
	4	Consensus	GTTTCAATCCTTGTTTTAATGGATCTTGCTCTCGAAT	**-2.40**	**-40.50**	**-122.8**	**56.5**	**-3.33**	**14222**	**-54.16**
	5	Consensus	GTTTCAATCCCTCTAAGGTCTGATTTTAAC	**-2.70**	**-55.80**	**-171.2**	**52.7**	**-3.11**	**979**	**-560.37**
		Variant	GTTTCAATCCCTCAAAGGTCTGATTTTAAC	**-2.70**	**-55.80**	**-171.2**	**52.7**	**-3.11**	**1096**	
***Ne.* SR1**	1	Consensus	GTTTCAATCCTTGTTGTGGTGGATTCGTCTTTCAGAC	**-5.50**	**-55.20**	**-160.2**	**71.3**	**-4.99**	**31397**	-
***Nca.* SCU2**	1	Consensus	CTTTCAATCCTACTATAGTTCGATTCAAAC	**-2.90**	**-63.90**	**-196.6**	**51.7**	**-1.56**	**416**	**-1624.82**
	2	Consensus	AGGTTTGAGGAGGCTATAGCATGCTTTGATAAGGCTATAGAGAT	**-7.70**	**-69.00**	**-197.6**	**75.9**	-8.92	**328303**	**-171.62**
		Variant	AGGTTTGAGGAGGCTATAGCATGCTTTGATAGAGCGTTAAAGAT	**-7.90**	**-84.80**	**-247.9**	**68.8**	**-8.31**	**414089**	
		Variant	ATGCTGAACGAGGCTGTAAAGTGCTTCAACAGGGCTATAGAGAT	**-7.20**	**-84.60**	**-249.5**	**65.8**	**-9.33**	**75338**	
	3	Consensus	ATTTCAATCCTACTATAGTTCGATTCAAAC	**-3.10**	**-64.40**	**-197.6**	**52.6**	**-1.53**	**475**	**-1615.77**
		Variant	CTTTCAATCCTACTATAGTTCGATTCAAAC	**-2.90**	**-63.90**	**-196.6**	**51.7**	**-1.56**	**416**	

***Ap.* K1**	1	Consensus	GAATCTTCGAGATAGAATTGCAAG	**-1.80**	**-32.30**	**-98.3**	**55.3**	**-1.30**	**161**	**-619.81**
		Variant	GAATCT**C**CGAGATAGAATTGCAAG	**-1.80**	**-32.30**	**-98.3**	**55.3**	**-0.76**	**102**	
	2	Consensus	GCATATCCCTAAAGGGAATAGAAAG	**-5.70**	**-51.20**	**-146.7**	**75.8**	**-4.93**	**78**	**-1078.02**
	3	Consensus	CTTGCAATTCTATCTCGAAGATTC	**-1.10**	**-33.30**	**-103.8**	**47.5**	**-0.74**	**81**	**-496.93**
		Variant	CTTGCAATTCTATCTCG**G**AGATTC	**-2.70**	**-43.90**	**-132.8**	**57.3**	**-0.88**	**124**	
***Ih.* KIN4/I**	1	Consensus	CTTTCTATCCTCTCTTGAGACTCCAC	**-2.00**	**-27.80**	**-83.1**	**61**	**-2.55**	**43**	**-151.99**
		Variant	CTTT**T**CATCCTCT**T**TTGAGACTCCAC	**-2.10**	**-35.80**	**-108.6**	**56.3**	**-2.93**	**56**	
	2	Consensus	CTTTCTATCCTCTTTTGAGACTCCAC	**-1.70**	**-36.90**	**-113.4**	**51.9**	**-2.55**	**45**	**-255.24**
		Variant	CTTTC**C**ATCCTCT**C**TTGAGACTC**T**AC	**-2.00**	**-27.80**	**-83.1**	**61**	**-2.55**	**43**	
		Variant	CTTTCTATCCTCT**C**TTGAGACTCC**G**C	**-2.00**	**-27.80**	**-83.1**	**61**	**-2.55**	**43**	
	3	Consensus	GAGTCTCAAAAGAGGATAGAAAG	**-1.70**	**-36.90**	**-113.4**	**51.9**	**-2.65**	**34**	**-164.09**
		Variant	GAG**A**CTCAA**G**AGAGGATAGAAAG	**-2.30**	**-40.00**	**-121.5**	**55.9**	**-2.04**	**12**	
	4	Consensus	CTTTCTATCCTCTTTTGAGACTCCAC	**-1.70**	**-36.90**	**-113.4**	**51.9**	**-2.55**	**45**	**-79.21**
		Variant	CTTTC**CGC**CCTCT**CC**TGAGACTCCAC	**-1.70**	**-36.90**	**-113.4**	**51.9**	**-2.53**	**35**	
	5	Consensus	CTTTCTATCCTCTTTTGAGACTCCAC	**-1.70**	**-36.90**	**-113.4**	**51.9**	**-2.55**	**45**	**-267.35**
		Variant	CTTTCTAT**T**CTCTTTTGAGACTCCAC	**-3.30**	**-44.30**	**-132.1**	**61.9**	**-3.71**	**54**	
		Variant	CTTTC**C**AT**T**CTCT**C**TTGAGACTCCAC	**-3.30**	**-44.30**	**-132.1**	**61.9**	**-3.71**	**50**	
	6	Consensus	GTGGAGTCTCAAAAGAGGATAGAAAG	**-3.60**	**-56.00**	**-168.9**	**58.3**	**-2.66**	**81**	**-217.03**
		Variant	GTAGAGTCTCAA**G**AGAGGATAGAAAG	**-3.40**	**-60.10**	**-182.8**	**55.5**	**-2.67**	**80**	
	7	Consensus	GAGTCTCAAGAGAGGATGGAAAG	**-2.90**	**-41.40**	**-124.1**	**60.3**	**-2.59**	**44**	**-175.20**
		Variant	GAGTCTCAA**A**AGAGGATAGAAAG	**-2.90**	**-41.40**	**-124.1**	**60.3**	**-2.65**	**34**	
	8	Consensus	GTGGAGTCTCAAGAGAGGATAGAAAG	**-3.40**	**-60.10**	**-182.8**	**55.5**	**-2.66**	**84**	**-190.40**
		Variant	GTGGAGTCTCAAGAGAG**A**AT**G**GAAAG	**-3.80**	**-50.00**	**-148.9**	**62.5**	**-3.22**	**80**	
		Variant	GTGGAGT**T**TCA**G**GA**A**AG**A**AT**G**GAAAG	**-2.60**	**-34.90**	**-104.1**	**61.9**	**-2.08**	**144**	
		Variant	**A**T**A**GAG**C**CT**T**AAG**G**GAGGATAGAGAG	**-4.40**	**-56.10**	**-166.6**	**63.3**	**-3.11**	**80**	
	9	Consensus	GATTAAACGAAAGAGAACTGCAAG	**0.60**	**-20.90**	**-69.3**	**28.3**	**-0.05**	**29**	**-443.48**
		Variant	GATTAAAC**A**AAAGAGAACTGCAAG	**1.10**	**-18.60**	**-63.5**	**19.6**	**-0.06**	**29**	
		Variant	GATTAAACGAAAGAGAACTGCA**G**G	**0.60**	**-20.90**	**-69.3**	**28.3**	**-0.05**	**29**	
		Variant	GATTAAACGA**T**AGA**A**AAG**C**G**T**AAG	**-1.90**	**-29.50**	**-88.9**	**58.3**	**-0.48**	**35**	
	10	Consensus	CTTTCTATCCTCTTTTGAGACTTCAC	**-1.70**	**-36.90**	**-113.4**	**51.9**	**-2.56**	**47**	**-158.54**
		Variant	CTTTCTATCCTCT**C**TTGAGACTTCAC	**-2.00**	**-27.80**	**-83.1**	**61**	**-2.56**	**45**	
***Ia.* DSM 17230**	1	Consensus	CTTTCAATTCCATATATTGGATTC	**-4.20**	**-42.20**	**-122.5**	**71.2**	**-3.81**	**131**	**-207.30**
	2	Consensus	GAATCCTATAAATGGAATTGAAAG	**-2.90**	**-44.90**	**-135.4**	**58.4**	**-2.47**	**87**	**-204.63**
		Variant	GAATCCTA**C**AAATGGAATTGAAAG	**-2.90**	**-44.90**	**-135.4**	**58.4**	**-2.47**	**76**	
	3	Consensus	CTTTCAATTCCATATATTGGATTC	**-4.20**	**-42.20**	**-122.5**	**71.2**	**-3.81**	**131**	**-383.57**
		Variant	CTTTCAATTCCAT**T**TAT**A**GGATT**T**	**-2.40**	**-38.70**	**-117**	**57.5**	**-1.94**	**179**	
	4	Consensus	CTTTCTACTCCCTTTTGGGAGTTTC	**-8.50**	**-66.30**	**-186.3**	**82.6**	**-9.90**	**249**	**-580.47**
		Variant	**AT**CTT**TCTA**CC**TC**TTTGGGAGTTTC	**-2.70**	**-37.20**	**-111.2**	**61.2**	**-2.92**	**248**	
	5	Consensus	GAAACTCCCAAAAGGGAGTAGAAAG	**-9.40**	**-71.50**	**-200.2**	**83.9**	**-9.75**	**57**	**-587.10**
	6	Consensus	GAAACTCCCAAAAGGGAGTAGAAAG	**-9.40**	**-71.50**	**-200.2**	**83.9**	**-9.75**	**57**	**-819.98**
	7	Consensus	GAATCCAATAAATGGAATTGAAAG	**-4.90**	**-51.10**	**-148.9**	**69.8**	**-3.14**	**75**	**-280.45**
		Variant	GAATCCAATAAATGGAATTGAA**G**G	**-4.90**	**-51.10**	**-148.9**	**69.8**	**-3.14**	**104**	
***Hb.* DSM 5456**	1	Consensus	GAACAACTCAAAAGAGAATTGCAAG	**-2.10**	**-41.00**	**-125.4**	**53.7**	**-2.03**	**64**	**-1017.74**
		Variant	GAACAACTCAAAAGAGAATTGCA**GA**	**-2.10**	**-41.00**	**-125.4**	**53.7**	**-2.04**	**70**	
		Variant	GAA**T**AACTCAAAAGAGAATTG**T**AA**T**	**-2.10**	**-41.00**	**-125.4**	**53.7**	**-1.95**	**139**	
	2	Consensus	CTTGCAATTCTCTTTTGAGTTGTTC	**-6.00**	**-61.20**	**-177.9**	**70.7**	**-6.67**	**196**	**-1163.26**
		Variant	CTT**A**CAATTCTCTTTTGAGTTGTTC	**-5.40**	**-59.00**	**-172.8**	**68.2**	**-6.40**	**196**	

***Pd.* Su06**	1	Consensus	GCATAACTCAAAAGAGAATTGTAAG	**-3.40**	**-67.30**	**-206**	**53.5**	**-3.24**	**130**	**-1224.19**
		Variant	**A**CA**C**AACTCAAAAGA**CG**A**C**TGTAAG	**-0.80**	**-57.60**	**-183.1**	**41.3**	**-0.47**	**46**	
	2	Consensus	CTTACAATTCTCTTTTGAGTTATGC	**-2.20**	**-41.80**	**-127.6**	**54.2**	**-2.96**	**167**	**-1178.38**
		Variant	**TTAGT**AATTCTC**CG**TTGAGT**C**A**A**GC	**-0.70**	**-30.20**	**-95.1**	**44.3**	**-1.70**	**270**	
	3	Consensus	CTTTCAGTTCTATTTGTTATGATTC	**-0.80**	**-30.10**	**-94.4**	**45.4**	**-0.41**	**135**	**-194.50**
		Variant	C**A**TTCAGTTCTATTTG**CC**ATGATTC	**-0.80**	**-30.10**	**-94.4**	**45.4**	**-0.64**	**157**	
	4	Consensus	GAATCATAACAAATAGAACTGAAAG	**-0.40**	**-32.50**	**-103.4**	**40.8**	**-0.11**	**24**	**-171.20**
		Variant	GAATCATAACAAATAGAACTG**G**AAG	**0.30**	**-29.00**	**-94.4**	**33.8**	**-0.12**	**30**	
	5	Consensus	GAATCATAACAAATAGAACTGAAA	**-0.40**	**-32.50**	**-103.4**	**40.8**	**-0.11**	**17**	**-216.47**
		Variant	GAATCATAACAAATAGAA**T**TGAAA**A**	**-0.40**	**-32.50**	**-103.4**	**40.8**	**-0.33**	**61**	
***Pf.* 1A**	1	Consensus	GAAACAACCAAGAATGAATTGAAAG	**-0.60**	**-27.90**	**-88**	**43.8**	**-0.38**	**28**	**-727.25**
	2	Consensus	CTTTCAATTCATTCTTTGTTGTTTC	**-0.10**	**-29.70**	**-95.4**	**38**	**-0.50**	**35**	**-1425.58**
		Variant	**TC**TTCAAT**C**C**G**TTCTT**G**GTTGTTTC	**-1.70**	**-53.90**	**-168.3**	**47.1**	**-2.62**	**99**	
	3	Consensus	GAAACAACCAAGAATGAATTGAAAG	**-0.60**	**-27.90**	**-88**	**43.8**	**-0.38**	**28**	**-419.72**
		Variant	GAAACAACCAAGAATGAATTGAA**GA**	**-0.60**	**-27.90**	**-88**	**43.8**	**-0.38**	**28**	
	4	Consensus	CTTTCAATTCATTCTTGGTTGTTTC	**-0.70**	**-28.00**	**-88**	**44.9**	**-1.30**	**99**	**-1829.01**
	5	Consensus	GAAACAACCAAGAATGAATTGAAAG	**-0.60**	**-27.90**	**-88**	**43.8**	**-0.38**	**28**	**-665.32**
	6	Consensus	CTTTCAATTCATTCTTGGTTGTTTC	**-0.70**	**-28.00**	**-88**	**44.9**	**-1.30**	**99**	**-140.15**
		Variant	**TC**T**C**CAATTCATTCTTGGTTGTTTC	**-2.10**	**-37.60**	**-114.4**	**55.3**	**-1.89**	**99**	
	7	Consensus	CTTTCAATTCATTCTTGGTTGTTTC	**-0.70**	**-28.00**	**-88**	**44.9**	**-1.30**	**99**	**-434.92**
***Ss.* SULA**	1	Consensus	GATTAATCCCAAAAGGAATTGAAAG	**-3.40**	**-47.80**	**-143.1**	**60.7**	**-3.10**	**228**	-
	2	Consensus	CTTTCAATTCCTTTTGGGATTAATC	**-4.50**	**-47.70**	**-139.2**	**69.3**	**-5.11**	**395**	**-1186.28**
		Variant	CTTTCAATTCCTTTT**A**GGATTAATC	**-3.60**	**-36.00**	**-104.4**	**71.4**	**-2.56**	**288**	
	3	Consensus	GATAATCTCTTATAGAATTGAAAG	**-1.20**	**-28.10**	**-86.7**	**50.8**	**-0.61**	**228**	-
		Variant	**T**ATAATCTCTTATAGAATTGAAAG	**-1.20**	**-28.10**	**-86.7**	**50.8**	**-0.73**	**176**	
	4	Consensus	GATAATCTACTATAGAATTGAAAG	**-1.20**	**-28.10**	**-86.7**	**50.8**	**-0.88**	**122**	**-125.55**
***Sa.* HS-1**	1	Consensus	GTTTCAAGCCCTCAAAGGTAAGCTACAAAC	**-3.10**	**-47.30**	**-142.5**	**58.7**	**-3.68**	**718**	-
	2	Consensus	GTTTCAAGCCCTCAAAGGTAAGCTACAAAC	**-3.10**	**-47.30**	**-142.5**	**58.7**	**-3.68**	**718**	**-377.30**
***Si.* L.S.2.15**	1	Consensus	GATAAATCCCCAAAGGGATTGAGAG	**-7.00**	**-61.00**	**-174.1**	**77.2**	**-7.10**	**207**	**-129.65**
		Variant	G**GAT**AATCCCCAAAGGGATTGAGAG	**-7.80**	**-65.60**	**-186.3**	**78.8**	**-7.48**	**254**	
		Variant	GATAAATCCCCAAAGGGATTGA**A**AG	**-7.00**	**-61.00**	**-174.1**	**77.2**	**-7.10**	**196**	
	2	Consensus	CTTTCAATTCTATAGTAGATTAAC	**-1.80**	**-31.90**	**-97**	**55.5**	**-0.79**	**186**	-
		Variant	CTTTCAATTCTATAGTAGAT**C**AAC	**-1.80**	**-31.90**	**-97**	**55.5**	**-0.73**	**122**	
		Variant	CTTTCAATTCTATAGTAGATTA**G**C	**-1.80**	**-31.90**	**-97**	**55.5**	**-0.88**	**247**	
	3	Consensus	GTTAATCTACTATAGAATTGAAAG	**-2.10**	**-34.40**	**-104.1**	**57.1**	**-1.60**	**249**	**-1531.27**
		Variant	GTTAATCTA**T**TATAGAATTGAAAG	**-2.10**	**-34.40**	**-104.1**	**57.1**	**-1.61**	**394**	
		Variant	GTTAATCTACTATAGAATTGAA**GT**	**-2.10**	**-34.40**	**-104.1**	**57.1**	**-1.61**	**321**	

***Tt.* Kra1**	1	Consensus	CCTTCAAATCTCTATCTGAGATTC	-5.30	-55.80	-162.8	69.5	-5.65	81	-590.74
	2	Consensus	AGTGGAAATCAAAAGATAGTAGAAAC	-0.70	-35.00	-110.5	43.3	-0.48	42	-172.08
		Variant	**G**GTGGAAATCAAAAGATAGTAGAAAC	-3.00	-26.10	-74.4	77.2	-0.93	46	
		Variant	**C**GTGGAAATCAAAAGATAGTAGAAAC	-0.70	-35.00	-110.5	43.3	-0.48	42	
		Variant	GGTGGAAATCAAAAGATA**TGTAG**AA**A**	-3.00	-26.10	-74.4	77.2	-0.93	46	
		Variant	**A**GTGGAAATCAAAA**T**ATAGTAGAAAC	-0.60	-14.20	-43.8	50.6	-0.12	65	
	3	Consensus	CTTTCTACTATCTTTTGATTTCCAC	0.20	-29.80	-96.7	34.9	-0.53	10	-207.34
		Variant	**CCGTT**TACTATCTTT**C**GATTTCCA**T**	-0.10	-28.60	-91.8	38	-0.28	19	
		Variant	**G**TTTCTACTATCTTTTGATTTCCAC	0.20	-29.80	-96.7	34.9	-0.61	30	
		Variant	**A**TTTCTACTATCTTTTGATTTCCAC	0.20	-29.80	-96.7	34.9	-0.53	16	
	4	Consensus	CTTTCAATCCTCTCTTTGAGATTC	-3.00	-43.20	-129.6	60.1	-3.66	124	-747.32
		Variant	CTTTCAATCCTCT**T**TTTGAGATTC	-3.00	-43.20	-129.6	60.1	-3.62	163	
	5	Consensus	GAATCTCAAAGAGAGGATTGAAAG	-3.90	-47.30	-139.9	64.8	-3.04	168	-784.53
	6	Consensus	CTTTCAATCCTCTTTTTGAGATTC	-3.00	-43.20	-129.6	60.1	-3.62	163	-563.94
		Variant	CTTTCAATC**T**TCT**A**TTT**A**AGA**C**TC	-1.50	-30.70	-94.1	52.9	-0.77	75	
		Variant	CTTTCAATCCTC**AAC**TTGAGATTC	-3.10	-38.80	-115.1	63.9	-3.13	110	
	7	Consensus	GAATCTCAAAGAGAGGATTGAAAG	-3.90	-47.30	-139.9	64.8	-3.04	168	-396.60
***Vm.* 768-28**	1	Consensus	GATATTCTCTAAAGAGAATAGAAGT	-6.80	-68.70	-199.5	71	-5.99	373	-737.35
		Variant	GATATTCTCTAAAGAGAATAGAAG**G**	-6.80	-68.70	-199.5	71	-5.99	244	
		Variant	GAT**G**TT**TT**CT**G**AAGAG**G**AT**G**GA**G**G**G**	-3.20	-43.40	-129.6	61.6	-2.44	538	
	2	Consensus	CTTTCAATATTCTATTGAAATCAAC	-5.40	-59.70	-175	67.8	-4.38	236	-265.88
		Variant	CTTTCAA**C**ATTCTATTGAAATCAAC	-3.20	-48.50	-146	58.9	-2.03	116	
	3	Consensus	CTTTCAATATTCTATTGAAATCAAC	-5.40	-59.70	-175	67.8	-4.38	236	-332.72
		Variant	**ACA**TCAA**A**ATTC**CTCG**GAAATCAAC	-0.40	-30.50	-97	41.1	-1.38	30	
		Variant	C**C**TTCAA**C**ATTCTATTGAAATCAAC	-2.50	-44.50	-135.4	55.4	-1.42	64	
		Variant	CTTTCAA**C**ATTCTATTGAAATCAAC	-3.20	-48.50	-146	58.9	-2.03	116	
	4	Consensus	TTTCAATATTCTTTTGAAATCAAC	-3.20	-49.20	-148.3	58.5	-2.83	155	-114.97
		Variant	TTTCAA**C**ATTCTTTT**T**AAA**AATCA**	0.80	-18.30	-61.5	24	-0.03	113	
		Variant	TTTCAA**C**ATTCTTTTGAAATCAAC	-3.10	-48.30	-145.7	58.2	-1.99	137	

The values of ΔG, ΔH, ΔS, and Tm, referring to Gibbs energy, enthalpy, entropy, and degradation temperature, respectively, were calculated using MFold, while the MFE DR, folding kinetics, and MFE CRISPR were calculated using RNAFold.

Table S4. Thermodynamics of direct repeats (DR) in Bacteria

Species	CRISPR	Sequence type	DR Sequence (5'-3')	ΔG (kcal/mol)	ΔH (kcal/mol)	ΔS cal/(K·mol)	T_m (°C)	MFE DR (kcal/mol)	DR folding kinetics (structures)	MFE CRISPR (kcal/mol)
***Ab.* A388**	1	Consensus	GTTCATGGCGGCATACGCCATTTAGAAA	-9.60	-83.10	-236.9	77.5	-9.72	704	-1110.46
***Pa.* UCBPP-PA14**	1	Consensus	GTTCACTGCCGTATAGGCAGCTAAGAAA	-8.50	-96.80	-284.7	66.8	-8.77	436	-414.96
		Variant	GTTCACTGCCGTATAGGCA**TAGGCAGCT**	-7.40	-59.10	-166.6	81.3	-8.25	801	
		Variant	**T**TTCACTGCC**AC**ATAGG**TC**G**TC**AAGAAA	-4.70	-57.50	-170.2	64.6	-3.77	762	
	2	Consensus	TTTCTTAGCTGCCTACACGGCAGTGAAC	-10.40	-68.80	-188.2	92.2	-9.79	1084	-579.11
***Ec.* K-12**	1	Consensus	CGGTTTATCCCCGCTGGCGCGGGGAACTC	-14.20	-104.80	-292.1	85.6	-14.74	3314	-430.81
		Variant	CGGTTTATCCCCGCT**AA**CGCGGGGAACTC	-14.20	-104.80	-292.1	85.6	-14.74	2294	
		Variant	CGGTTTATCCCCGCTG**AT**GCGGGGAAC**A**C	-13.80	-86.10	-233.1	96.1	-15.14	1478	
	2	Consensus	GGTTTATCCCCGCTGGCGCGGGGAACAC	-14.10	-84.90	-228.2	98.7	-14.44	1560	-214.37
		Variant	GGTTTATCCCCGCTGGCGCGGGGAAC**T**C	-14.10	-84.90	-228.2	98.7	-14.56	3129	
***Pm.* FDAARGOS_67**	1	Consensus	CGGTTCATCCCCGTATACACGGGGAACAC	-12.10	-79.80	-218.2	92.4	-12.23	747	-144.61
		Variant	**TAA**TT**TA**TCCCCGTATACACGGGGAACAC	-12.10	-79.80	-218.2	92.4	-12.07	508	
	2	Consensus	CGGTTCATCCCCGTGCATACGGGGAACAC	-12.20	-80.30	-219.5	92.5	-12.23	904	-234.80
		Variant	CG**A**TTCATCCCCGT**A**CATACGGGGAACAC	-12.20	-79.40	-216.6	93.3	-12.07	363	
***Sm.* N4-5**	1	Consensus	GTGCACTGCCGTACAGGCAGCTTAGAAA	-8.40	-59.10	-163.4	88.3	-8.68	408	-252.55
		Variant	GTGCACTGCCGTACAGGCAG**AA**TA**T**A**GG**	-9.10	-63.70	-176	88.6	-8.78	270	
	2	Consensus	GTTCACTGCCGCATAGGCAGCTTAGAAA	-8.50	-96.80	-284.7	66.8	-8.71	524	-447.53
		Variant	GTTCACTGCCG**T**ATAGGCAGCTTAGAAA	-8.50	-96.80	-284.7	66.8	-8.71	631	
		Variant	GTTCACTGCCG**TCC**AGGCAG**T**TT**GA**A**T**A	-11.90	-95.40	-269.2	81.2	-11.84	760	
***Ecl.* C2-1**	1	Consensus	TTTCTAAGCTGCCTGTACGGCAGTGCAC	-10.40	-68.80	-188.2	92.2	-9.18	724	-435.81
		Variant	TT**G**CTAAGCTGC**A**T**T**T**G**C**A**GCAGTGC**G**C	-12.90	-96.60	-269.8	84.8	-10.93	1998	
	2	Consensus	TTTCTAAGCTGCCTGTACGGCAGTGAAC	-10.40	-68.80	-188.2	92.2	-9.59	1255	-281.18
		Variant	**GAAA**T**GT**GCT**A**CCTGTACGGCAGTGAAC	-4.40	-51.40	-151.5	66	-4.25	343	
***Kp.* AATZP**	1	Consensus	CGGTTTATCCCCGCTGGCGCGGGGAACAC	-14.10	-84.90	-228.2	98.7	-14.44	1601	-1293.61
		Variant	**G**GG**C**TTATCC**T**CGC**GAAT**GCGGGGAACAC	-12.00	-80.50	-220.8	91.3	-11.39	531	
		Variant	CGGTTTATCCCCGCT**T**GCGCGGGGAACAC	-14.10	-84.90	-228.2	98.7	-14.44	1271	
***Ef.* SRCM103470**	1	Consensus	GTTTTAGAGCTATGCTGATTTGAATGCTTCCAAAAC	-4.20	-60.40	-181.2	60.1	-4.22	23963	-192.86
		Variant	GTTTTAGAGCTATGCTGATTTGAAT**A**CT**A**C**TCT**A**GA**	-4.10	-71.60	-217.6	55.8	-4.55	14509	
***Efa.* OG1RF**	1	Consensus	GTTTTAGAGTCATGTTGTTTAGAATGGTACCAAAACT	-3.20	-47.20	-141.8	59.5	-3.14	31178	-179.46
		Variant	GTTTTAGAGTCATGTTGTTTAGAATGGTACCAAAA**CA**	-3.30	-70.40	-216.3	52.2	-3.42	29595	
		Variant	GTTTTAGAGTCATGTTGTTTAGAATGGTACCAAA**T**CT	-3.20	-47.20	-141.8	59.5	-2.67	28142	
		Variant	GTTTTAGAGTCATGTTGTTTAG**TT**T**TCGCAAATACGA**	-1.80	-66.30	-207.9	45.6	-1.57	37332	
	2	Consensus	GGTTTTGGTACCATTCTAAACAACATGACTCTAAAAC	-3.80	-83.40	-256.6	51.8	-3.86	7258	-151.38
		Variant	GGT**GA**T**A**GT**TTGT**TT**T**T**T**AACAACATG**G**CTCTAAAAC	-3.00	-74.30	-229.8	50	-5.28	18658	
		Variant	**A**GTTTTGGTACCATTCTAAACAACATGACTCTAAAAC	-4.00	-80.40	-246.3	53.2	-3.90	6802	
		Variant	**C**GTTTTGGTACCATTCTAAACAACATGACTCTAAAAC	-4.10	-78.80	-240.8	54	-3.98	6162	

***Se.* FDAARGOS_153**	1	Consensus	TGTTCTCGTCCCCTTTTCGGCGGGGTGGTTAGCGAAT	-7.60	-85.90	-252.4	67.1	-9.76	59998	-134.33
		Variant	**AA**T**A**CTCGTCCCCTTTT**TT**GCGG**A**GTGGTTA**T**CGAAT	-6.00	-69.00	-203.1	66.5	-4.77	16346	
		Variant	**A**GTTCTCGTCCCCTTTT**AT**GCGGGG**T**GTTA**T**CGAAT**T**	-7.90	-81.60	-237.6	70.2	-7.89	19698	
		Variant	**A**GTTCTCGTCCCCTTTT**AT**GCGGGGTGGTTA**T**CGAAT	-7.50	-81.60	-238.9	68.3	-8.48	27644	
	2	Consensus	GTTCTCGTCCCCTTTTCTTCGGGGTGGTTATCGATC	-7.30	-79.00	-231.1	68.5	-8.23	15832	-273.88
		Variant	**CACTCT**G**C**CCCCTTTTCTTCGGGGT**A**GTTATCGATC	-9.90	-67.40	-185.3	90.4	-10.96	4071	
		Variant	**A**TTCTCGTCCCCTTTTCTTCGGGGTGGTTATCGATC	-7.30	-79.00	-231.1	68.5	-8.22	15832	
***Hm.* NCTC12198**	1	Consensus	GTTTTAGCCACTTCATAAATATGTTTATGCTAAAAT	-6.30	-88.20	-264	60.8	-6.82	20182	-271.26
***Cj.* NS4-5-1**	1	Consensus	GTTTTAGTCCCTTTTTAAATTTCTTTATGGTAAAAT	-1.30	-80.60	-255.6	42	-0.87	26904	-134.10
		Variant	**A**TTTTAGTCCCTTTTTAAATTTCTTTATGGTAAAAT	-1.20	-82.30	-261.4	41.5	-0.81	26904	
***Sen.* SA19980677**	1	Consensus	CGGTTTATCCCCGCTGGCGCGGGGAACAC	-14.10	-84.90	-228.2	98.7	-14.44	1601	-322.44
		Variant	**GT**GTTTATCCCCGCTGGCGCGGGGAACA**T**	-14.40	-104.20	-289.5	86.7	-15.36	4640	
	2	Consensus	CGGTTTATCCCCGCTGGCGCGGGGAACAC	-14.10	-84.90	-228.2	98.7	-14.44	1601	-737.76
		Variant	CGGTTTATCCCCGCT**A**GCGCGGGGAACAC	-14.10	-84.90	-228.2	98.7	-14.44	1126	
		Variant	**AC**G**GC**TATCC**TT**G**T**TGGCGCGGGGAACAC	-8.10	-63.10	-177.3	82.6	-7.19	512	
***Nm.* NCTC10025**	1	Consensus	ATTGTAGCACTGCGAAATGAGAAAGGGAGCTACAAC	-8.90	-84.70	-244.3	73.4	-8.34	1633	-382.24
		Variant	ATTGTAGCACTGCGAAATGAGAA**T**GGG**C**GCTACAAC	-9.60	-99.50	-289.8	70.1	-9.41	3813	
***Hi.* NCTC11873**	1	Consensus	GTCGAAAGACATTGCCCTGTTCCAAAGGGATTGAGAC	-10.90	-89.10	-252.1	80.2	-8.55	19984	-204.72
		Variant	GTCGAAAGACATTGCCCTGTTCCAAAGGGATTGA**A**AC	-10.90	-89.10	-252.1	80.2	-8.55	14330	
***Sp.* MGAS23530**	1	Consensus	GTTTTAGAGCTATGCTGTTTTGAATGGTCCCAAAAC	-4.30	-46.20	-135	68.8	-5.58	22782	-156.63
		Variant	GTTTTAGAGCTATGCTGT**C**TTGAATGGTC**T**C**C**A**TT**C	-6.80	-76.30	-224	67.3	-5.85	13765	
***Sd.* CFSAN010956**	1	Consensus	TGTGTTCCCCGCGCCAGCGGGGATAAACCG	-15.10	-103.30	-284.3	90	-15.79	2956	-90.83
***Na.* NCTC12227**	1	Consensus	ATTGTAGCACTACGAGATGAGAGAGGAAGCTACAAC	-8.50	-92.50	-270.8	68.3	-6.96	1925	-161.07
***Vv.* YJ016**	1	Consensus	GTTTCAGACATGCCCGGTTTAGACGGGATTAAGAC	-7.20	-69.70	-201.5	72.7	-7.17	6364	-101.53
		Variant	GTTTCAGACATGCCCGGTTTAGACGGGAT**A**A**C**GA**A**	-7.20	-69.70	-201.5	72.7	-7.33	4246	
	2	Consensus	GTTTCAGACATGCCCGGTTTAGACGGGATTAAGAC	-7.20	-69.70	-201.5	72.7	-7.17	6364	-251.51
***Vp.* FORC_022**	1	Consensus	GTTAACTGCCACACAGGCAGCTTAGAAA	-8.50	-88.20	-256.9	70	-8.68	487	-567.17
		Variant	**T**TTAACTGCCACACAGGCAGCTTAGAAA	-9.40	-91.80	-265.6	72.3	-9.69	943	
		Variant	GTTAACTGCCACACAGGCAGCTTAGA**G**A	-8.50	-88.20	-256.9	70	-8.68	539	
***Vc.* FORC_076**	1	Consensus	GTTCACTGCCGCACAGGCAGCTTAGAAA	-8.50	-96.80	-284.7	66.8	-8.71	482	-1587.85
		Variant	GTTCACTGCCG**T**ACAGGCAGCTTAGAAA	-8.50	-96.80	-284.7	66.8	-8.71	571	
		Variant	GTTCACTGCCGCACAGGCAGCTTAG**TT**A	-8.40	-59.10	-163.4	88.3	-8.62	469	
***Bc.* KSM-K16**	1	Consensus	ATTTCAATCCACGCACTCACAAAGAGTGCGAC	-11.90	-83.90	-232.1	88.2	-11.53	449	-323.29
		Variant	ATT**CAT**ATCCACGCACTCACAAAGAGTGC**T**AC	-9.80	-73.60	-205.7	84.6	-9.40	214	
	2	Consensus	ATTTCAATCCACGCACTCACAAAGAGTGCGAC	-11.90	-83.90	-232.1	88.2	-11.53	449	-389.73
		Variant	ATTTCAATCCACGCACTCAC**C**AAAGAGTGCGA_	-11.60	-83.90	-233.1	86.7	-11.83	430	
	3	Consensus	ATTTCAATCCACGCACTCACATAGAGTGCGAC	-11.90	-83.90	-232.1	88.2	-11.53	449	-304.01
		Variant	ATTTCAATCCACGCACTCAC**TA**AGAGTGCG**G**C	-12.10	-82.90	-228.2	90	-12.02	667	
	4	Consensus	ATTTCAATCCACGCACTCATATAGAGTGCGAC	-11.90	-83.90	-232.1	88.2	-11.53	549	-460.84
		Variant	**ATCGATTGG**CACGCACTCATATAGAGTGCGAC	-11.90	-83.90	-232.1	88.2	-11.55	1348	
	5	Consensus	ATTTCAATCCACGCACTCATATAGAGTGCGAC	-11.90	-83.90	-232.1	88.2	-11.53	549	-220.95
		Variant	**CTGTTTT**TCCACG**A**A**G**TCATA**A**AGAGTGCGAC	-2.40	-57.10	-176.3	50.6	-2.33	3660	
	6	Consensus	ATTTCAATCCACGCACTCACTAAGAGTGCGAC	-11.90	-83.90	-232.1	88.2	-11.53	515	-192.43
		Variant	**GA**TTCAATC**G**ACGCACTC**G**C**AT**AGAGTGCGAC	-13.40	-110.60	-313.3	79.7	-12.59	1406	
		Variant	ATTTCAATCCACGCACTCAC**AT**AGAGTGCGAC	-11.90	-83.90	-232.1	88.2	-11.53	505	

***La.* YT1**	1	Consensus	GTTTTCTCCACGTATGTGGAGGTGATCCT	-11.00	-78.50	-217.6	87.5	-10.68	1601	-703.73
		Variant	GTTTTCTCCACGTATGTGGAGGTGATCC**C**	-11.00	-78.50	-217.6	87.5	-10.68	1539	
		Variant	GTTTTCTCCACGTATGTGGAGGTGATC**AA**	-11.00	-78.50	-217.6	87.5	-10.68	2022	
	2	Consensus	ATTTTCTCCACGTATGTGGAGGTGATCC	-10.50	-78.50	-219.2	84.8	-10.54	1426	-366.71
		Variant	ATTTTCTCCACG**A**A**A**GTGGAGGTGATCC	-12.70	-82.50	-225	93.4	-11.34	1978	
		Variant	ATTTT**TT**C**T**A**T**G**A**A**A**GTGGAGGT**A**A**C**CC	-6.90	-59.80	-170.5	77.4	-4.15	2631	
		Variant	ATTTTCTCCAC**AA**A**A**GTGGAGCT**ACATT**	-7.80	-68.30	-195	76.9	-8.53	693	
***Li.* Clip11262**	1	Consensus	GTTTTGTTAGCATTCAAAATAACATAGCTCTAAAAC	-5.10	-59.60	-175.7	66	-4.29	8760	-198.56
		Variant	**TGG**TT**T**TTAG**T**ATTC**G**AAATAGCATAGCTCTAAAAC	-1.80	-54.30	-169.2	47.6	-2.02	12785	
***Lm.* Lm3163**	1	Consensus	ATTTACATTTCATAATAAGTAGTTAAAAC	-0.90	-31.30	-98	46.1	-0.52	1639	-555.69
		Variant	**T**TT**ATG**ATTTCATAATAAGTAGTTAAAAC	-3.90	-46.60	-137.6	65.3	-1.91	2871	
		Variant	ATTTA**G**ATTTCATAATAAGTAGTTAAAAC	-0.90	-31.30	-98	46.1	-0.42	1737	
	2	Consensus	ATTTACATTTCACAATAAGTAACTAAAA	-0.70	-36.70	-116	43	-0.45	650	-108.05
	3	Consensus	GTTTTAGAGCTATGTTATTTTGAATGCTACCAAAAC	-4.00	-93.30	-287.9	50.8	-2.96	22344	-355.62
		Variant	GTTTTAGAGCTA**CA**TTATT**C**TGAAT**A**C**C**A**A**CA**C**A**TA**	-3.70	-44.20	-130.5	65.3	-3.95	4144	
***Mp.* 1049**	1	Consensus	GTTTTAGTACTATACTATTTTTAAGCAATACAAAAC	-2.70	-36.90	-110.2	61.4	-2.29	18832	-223.19
	2	Consensus	GTTTTAGTACTATACTATTTTTAAGCAATACAAAAC	-2.70	-36.90	-110.2	61.4	-2.29	18832	-139.04
		Variant	GTTTTAGTACTATACTATTTTTAAGCAATACAAAA**G**	-2.70	-36.90	-110.2	61.4	-2.27	20371	
		Variant	GTTTTAGT**G**CTATACTATTTT**A**A**TTTATTGAAATTA**	-4.90	-67.50	-201.8	61.2	-2.73	25306	
***Af.* DSM 20731**	1	Consensus	GTCGCCCCCGCAAGGGGGCGTGGATTGAAAT	-16.90	-89.40	-233.7	109.2	-16.49	2546	-2083.90
		Variant	GTCGCCCCCG**T**A**T**GGGGGCGTG**A**ATTGAAAT	-14.70	-85.40	-85.40	101.4	-15.69	2389	
		Variant	**GC**CCCCCGC**AAGG**GGG**A**GCGT**AA**ATTGAAAT	-14.90	-97.20	-265.3	93.1	-14.51	631	
***Bco.* HM-08**	1	Consensus	GTCGCTCCCTACATGGGGGCGTGGATTGAAAT	-12.00	-78.20	-213.4	93.2	-13.20	4076	-630.17
	2	Consensus	GTCGCTCCCTACATGGGGGCGTGGATTGAAAT	-12.00	-78.20	-213.4	93.2	-13.20	4076	-550.88
***Mc.* BBH18**	1	Consensus	TTTCTAAGCGACCTGTGCGGTCGTGAAG	-9.90	-68.00	-187.3	89.8	-9.73	1677	-110.03
		Variant	TTTCTAAGCGACCTGTGCGGTC**A**TGAAG	-6.40	-52.10	-147.3	80.4	-5.33	1114	
	2	Consensus	TTTCTAAGCGACCTGTGCGGTCGTGAAG	-9.90	-68.00	-187.3	89.8	-9.73	1677	-1181.55
		Variant	TT**G**CTAAGCGACCTGTGCGGTCGTGAAG	-9.90	-68.00	-187.3	89.8	-9.58	1362	
***Yp.* FDAARGOS_602**	1	Consensus	TTTCTAAGCTGCCTGTGCGGCAGTGAAC	-10.40	-68.80	-188.2	92.2	-9.63	1532	-170.10
		Variant	T**C**T**A**TAAGCTGCCTGTGCGGCAGTGAAC	-10.40	-68.80	-188.2	92.2	-9.22	644	

The values of ΔG, ΔH, ΔS, and Tm, referring to Gibbs free energy, enthalpy, entropy, and degradation temperature, respectively, were calculated using MFold, while the MFE DR, folding kinetics, and MFE CRISPR were calculated using RNAFold.

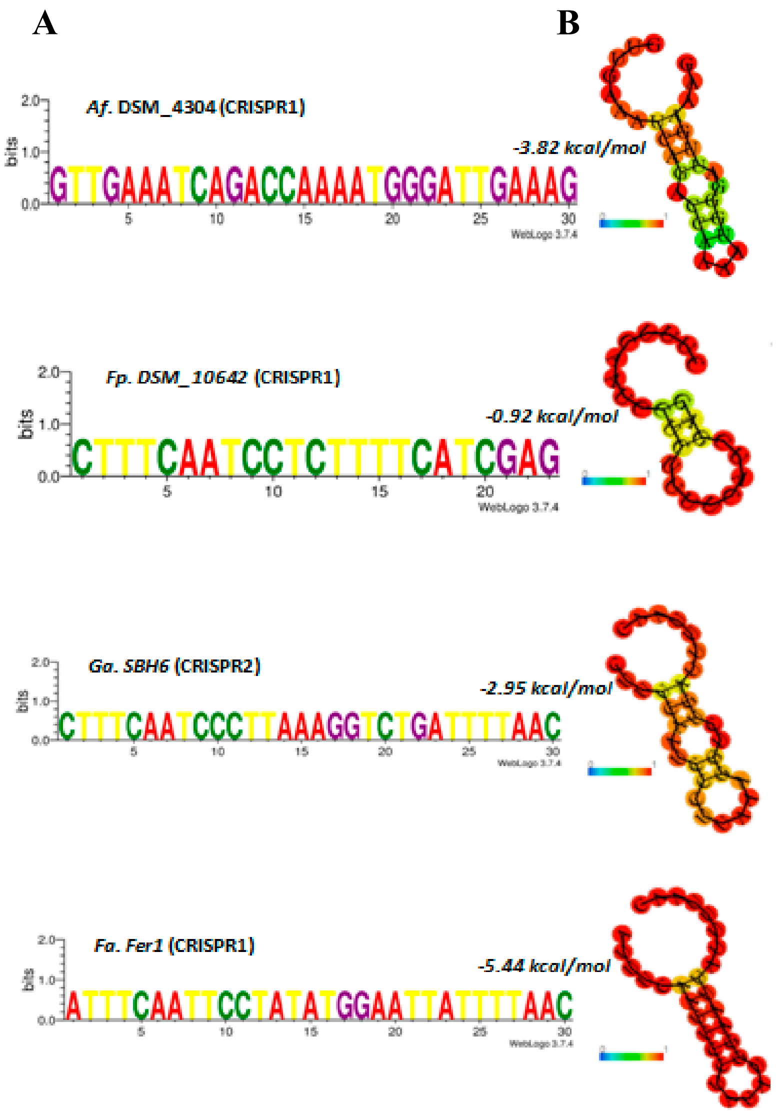
A
B
Af. DSM_4304 (CRISPR1)
GTTGAAATCAGACCAAAATGGGATTGAAAG
-3.82 kcal/mol
Fp. DSM_10642 (CRISPR1)
CTTTCAATCCTCTTTTCATCGAG
-0.92 kcal/mol
Ga. SBH6 (CRISPR2)
CTTTCAATCCCTTAAAGGTCTGATTTTAAC
-2.95 kcal/mol
Fa. Fer1 (CRISPR1)
ATTTCAATTCCTATATGGAATTATTTTAAC
-5.44 kcal/mol
bits
WebLogo 3.7.4

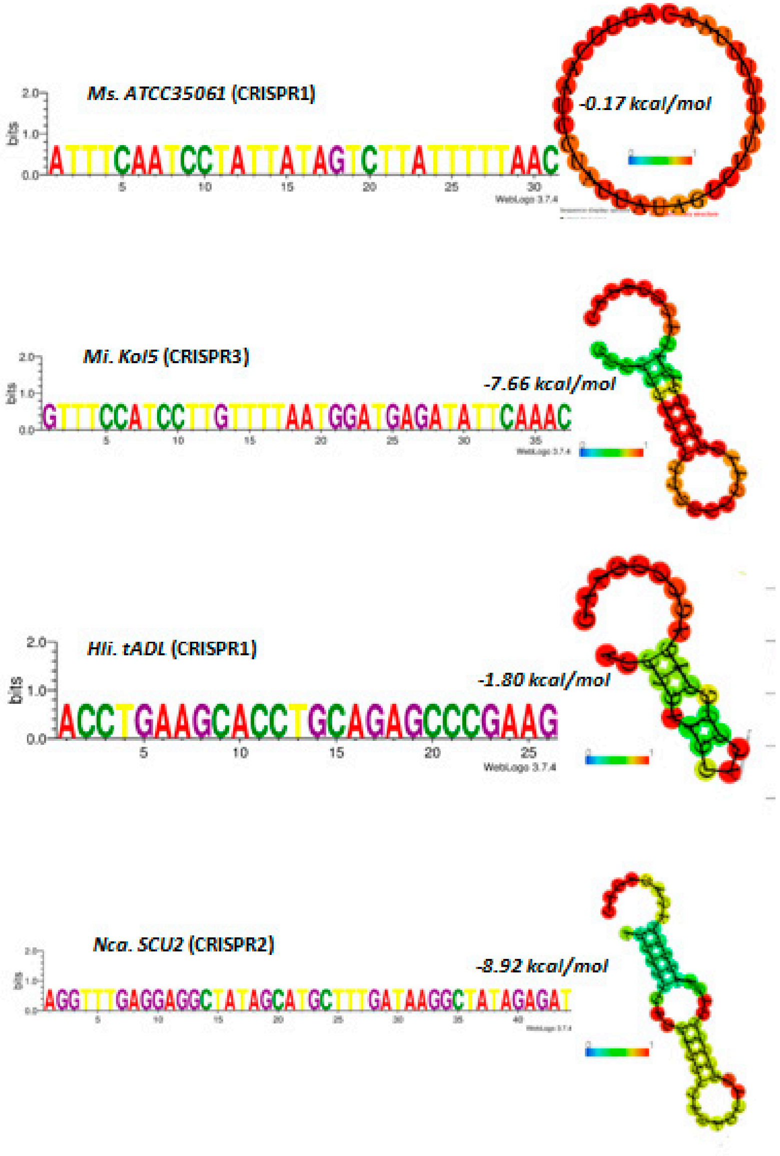

Figure S2. Representative RNA secondary structures of DRs in Archaea. A) Conservation of DRs represented with WebLogo 3.7.4. B) The RNA secondary structures and the minimum free energy (MFE) of formation of the direct repeats (DR) of some found CRISPRs. Typical

stem-loop stable structures consistently predicted for the DRs by RNAfold are shown. The darker base pairs represent the highest probability of pairing. Only structures that are different are shown.

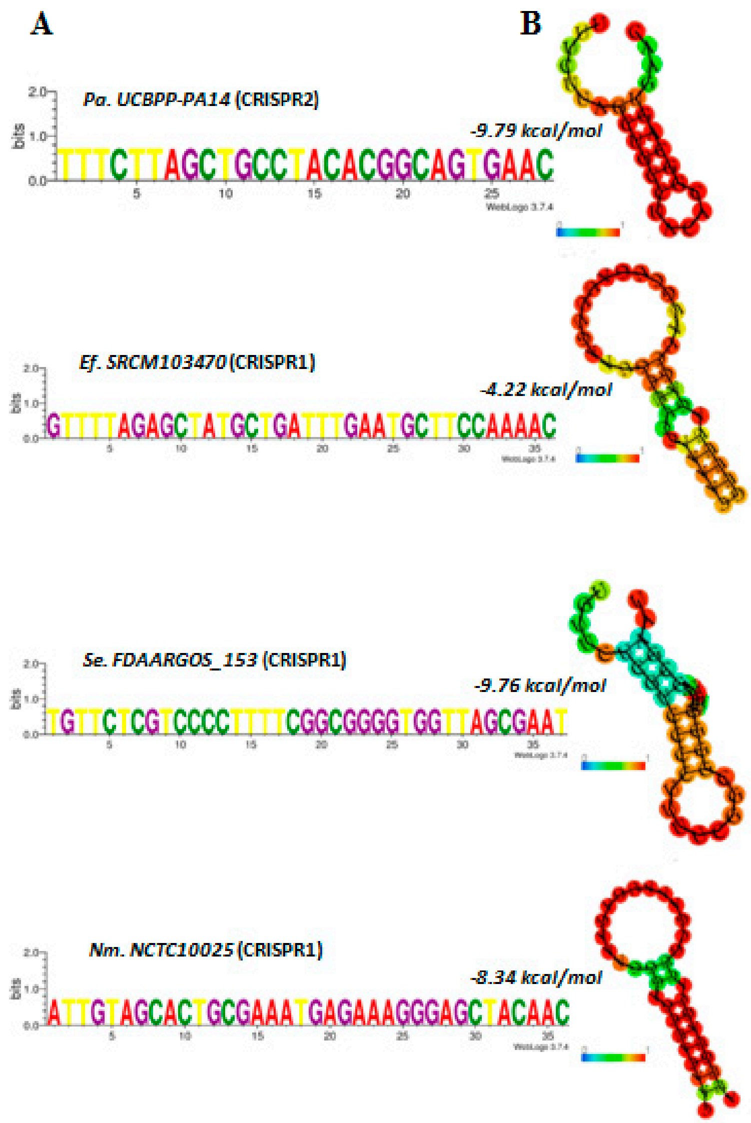
A
B
Pa. UCBPP-PA14 (CRISPR2)
-9.79 kcal/mol
TTTCTTAGCTGCCTACACGGCAGTGAAC
Ef. SRCM103470 (CRISPR1)
-4.22 kcal/mol
GTTTTAGAGCTATGCTGATTTGAATGCTTCCAAAAC
Se. FDAARGOS_153 (CRISPR1)
-9.76 kcal/mol
TGTTCTCGTCCCCTTTTCGGCGGGGTGGTTAGCGAAT
Nm. NCTC10025 (CRISPR1)
-8.34 kcal/mol
ATTGTAGCACTGCGAAATGAGAAAGGGAGCTACAAC

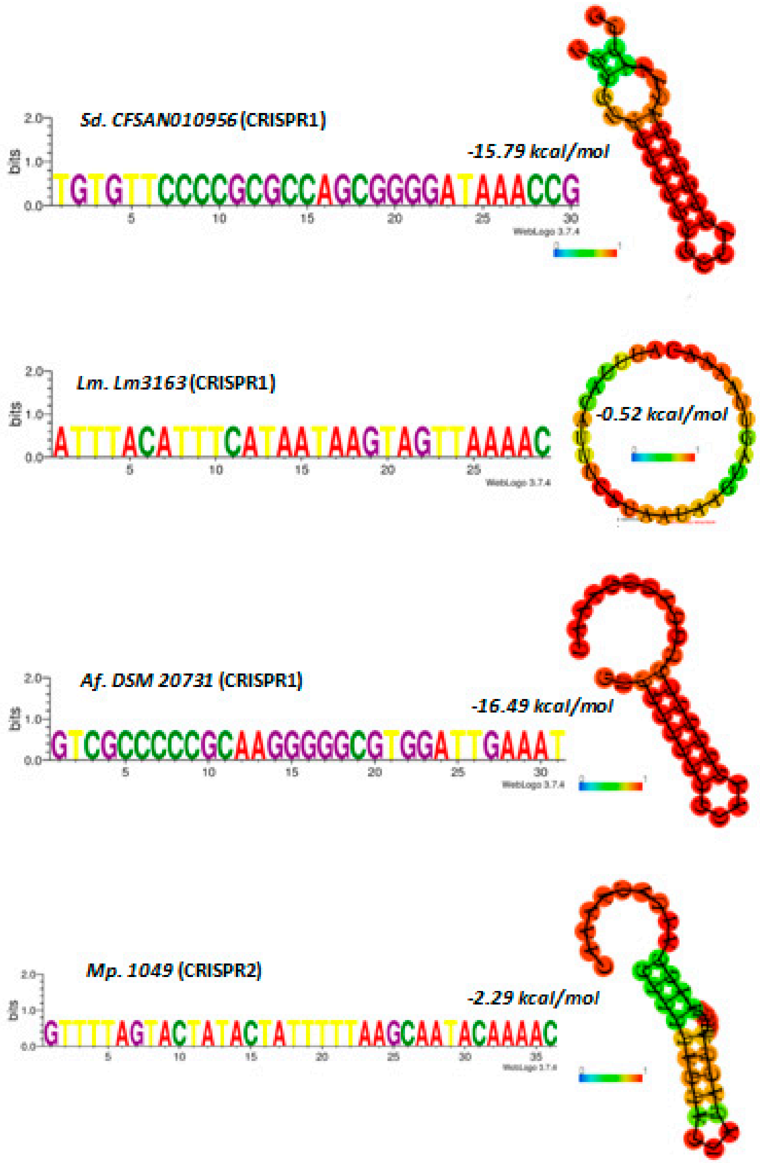

Figure S3. Representative secondary structures of RNA from direct repeats (DR) in Bacteria. A) Conservation of the DRs represented with WebLogo 3.7.4. B) The secondary RNA structures and the minimum free energy (MFE) of formation of the direct repeats (DR) from some found CRISPRs. The typical stem-loop stable structures predicted consistently for the DRs by RNAfold are shown. The darker base pairs represent the highest pairing probability. Only the structures that differ are shown.

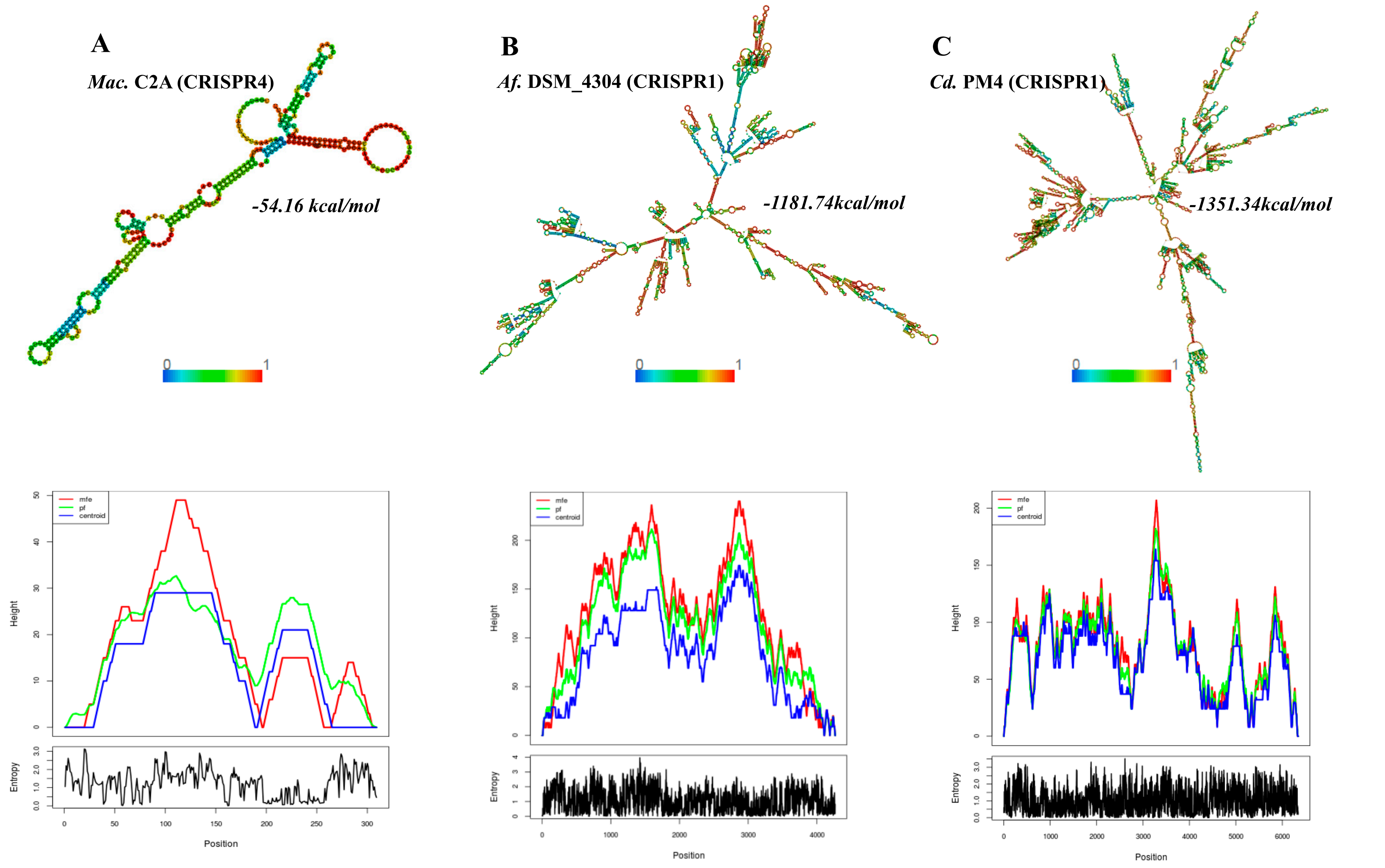

Figure S4. Secondary RNA structures of complete CRISPR arrays (DR + spacer) in Archaea. Only three structures with different MFEs from three representative species are shown. Additionally, entropy and energy levels at each position are displayed. A) CRISPR4 from *M. acetivorans* C2A with an MFE of -54.16 kcal/mol. B) CRISPR1 from *A. fulgidus* DSM_4304 with an MFE of -1,181.74 kcal/mol. C) CRISPR1 from *C. divulgatum* PM4 with an MFE of -1,351.34 kcal/mol.

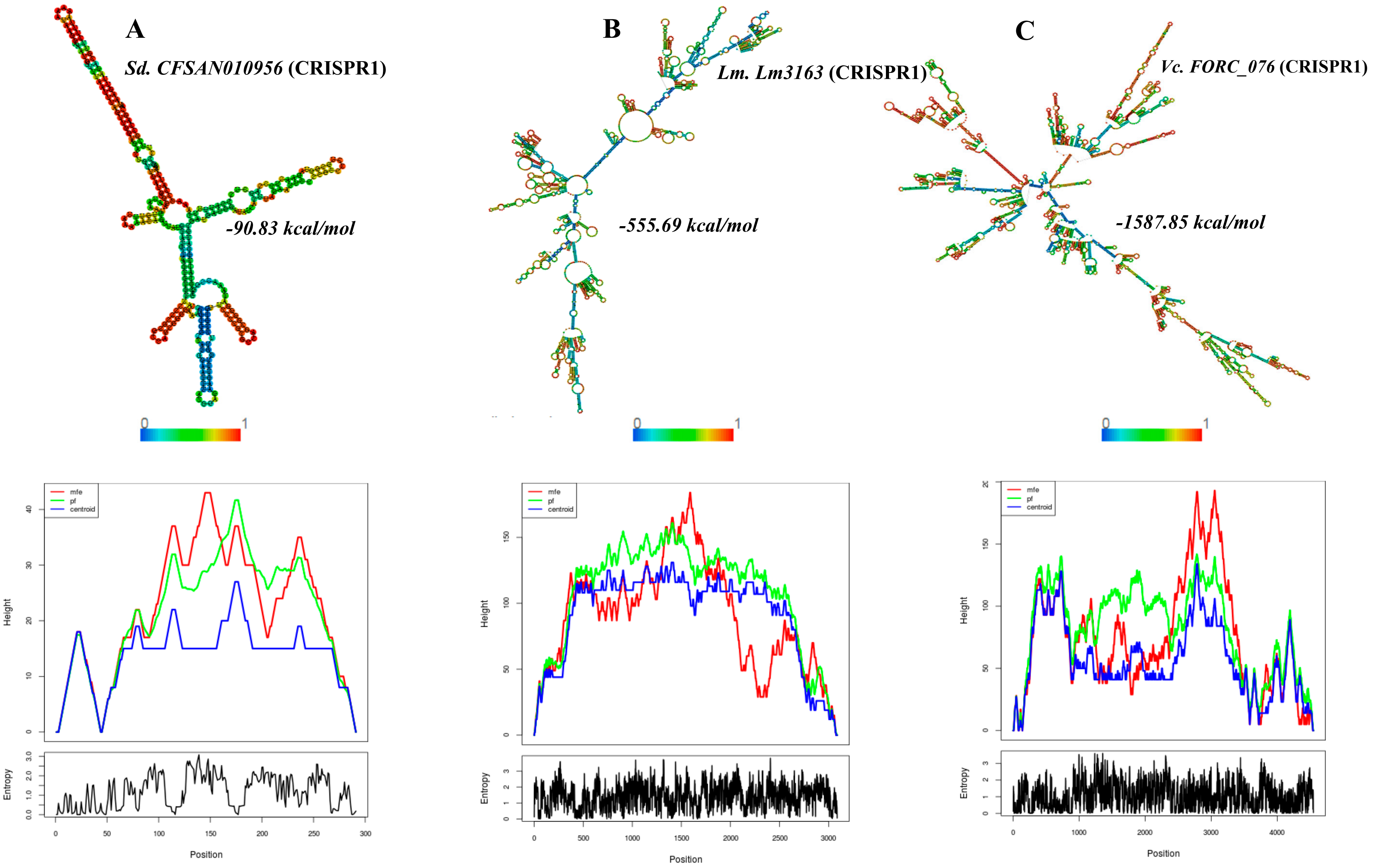

Figure S5. Secondary RNA structures of complete CRISPR arrays (DR + spacer) in Bacteria. Only three structures with different MFEs from three representative species are shown. Additionally, entropy and energy levels at each position are displayed. A) CRISPR1 from *S. dysenteriae* CFSAN010956 with an MFE of -90.83 kcal/mol. B) CRISPR1 from *L. monocytogenes* Lm3163 with an MFE of -555.69 kcal/mol. C) CRISPR1 from *V. cholerae* FORC_076 with an MFE of -1,587.85 kcal/mol.

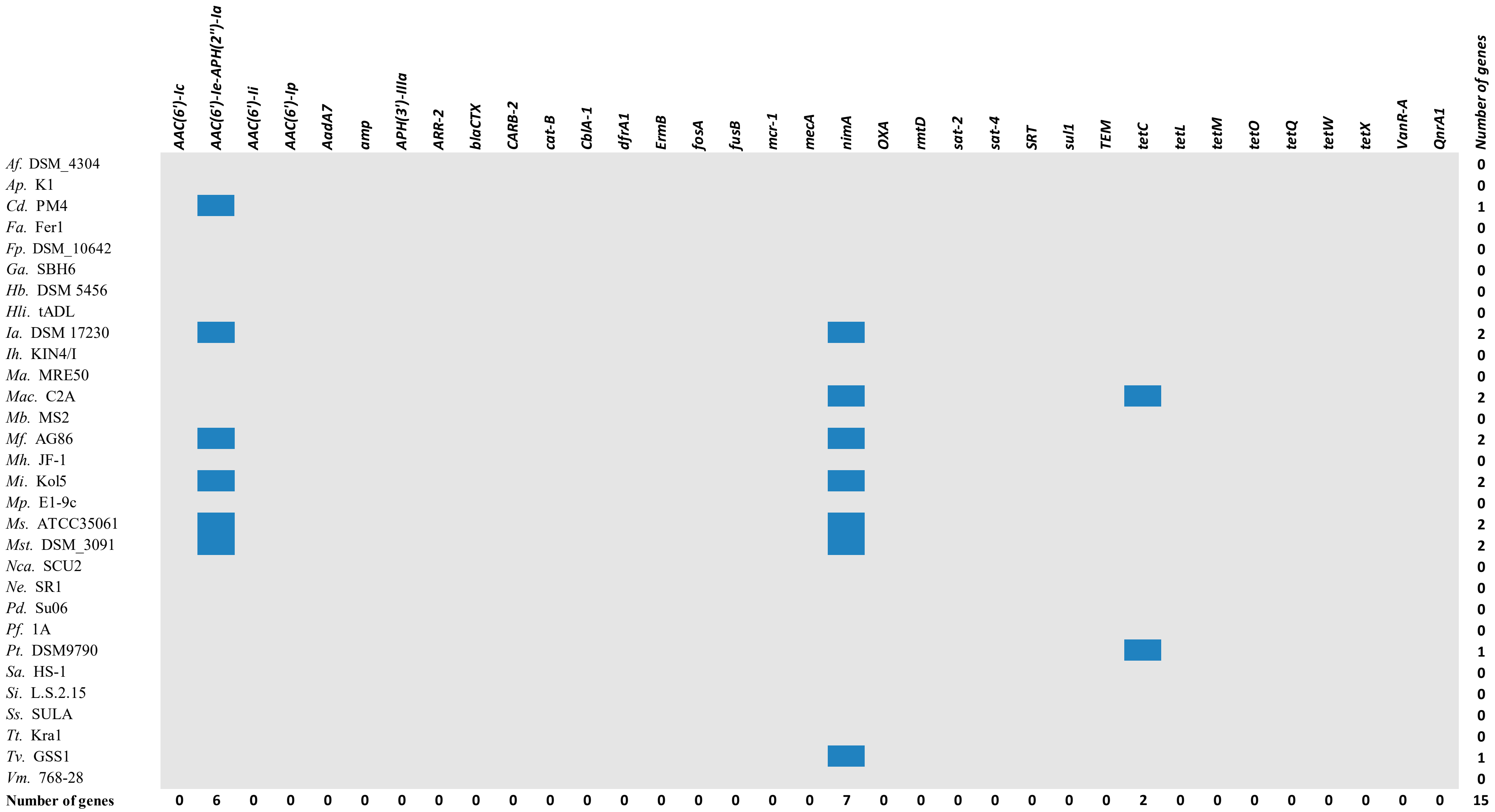

Figure S6. Antibiotic resistance genes in archaeal genomes with confirmed CRISPR-CAS systems.

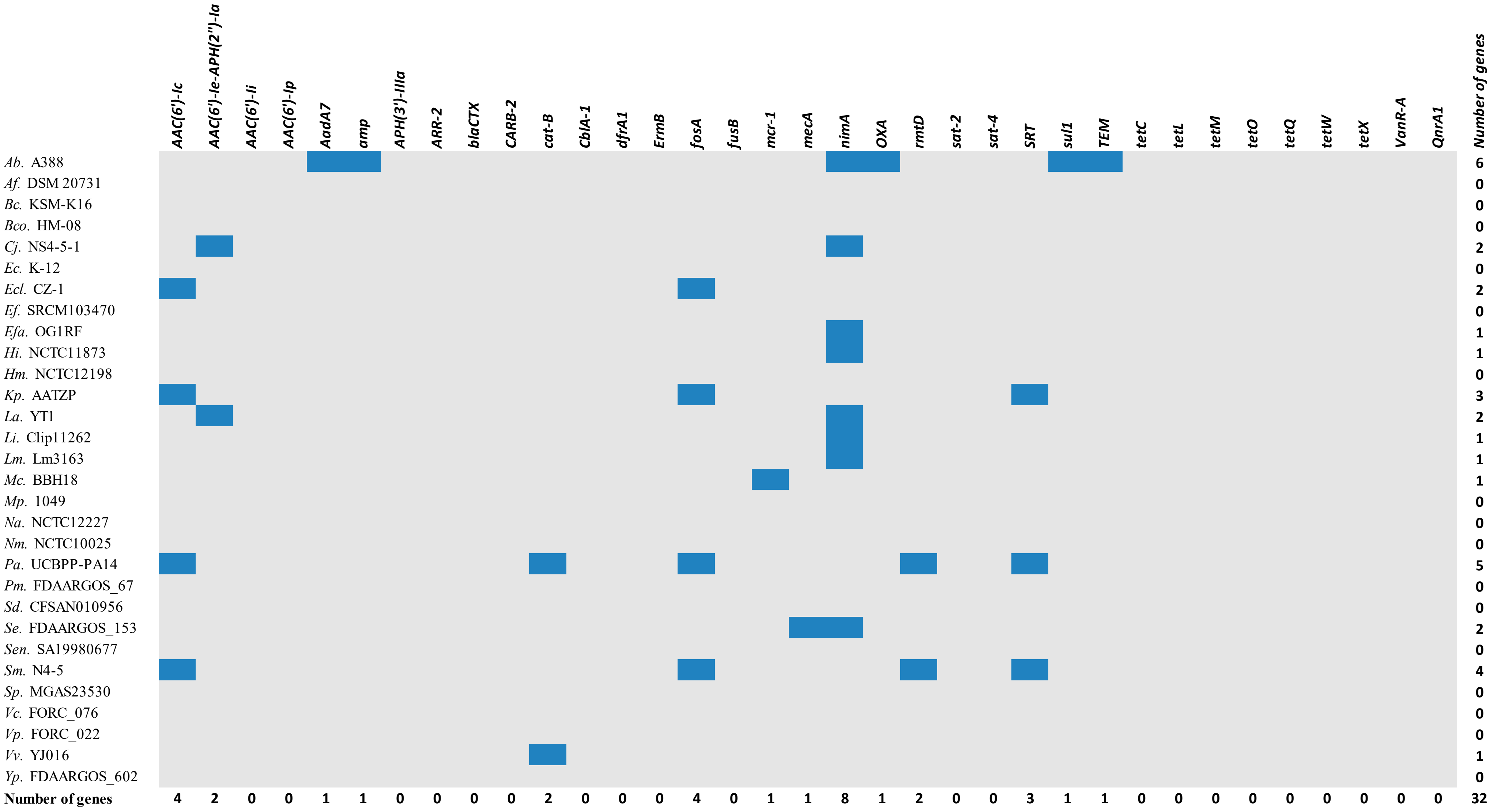

Figure S7. Antibiotic resistance genes in bacterial genomes with confirmed CRISPR-CAS systems.

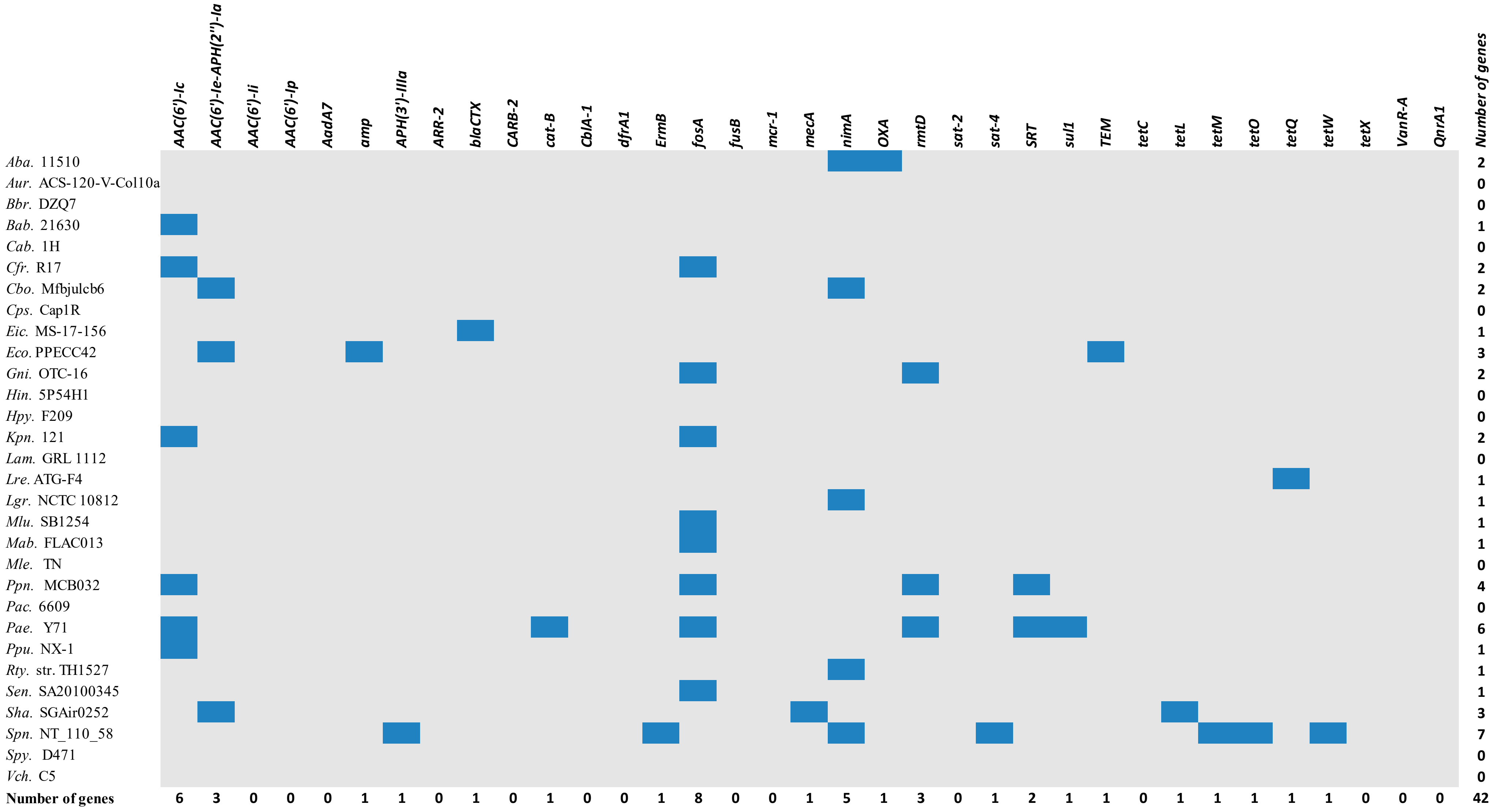

Figure S8. Antibiotic resistance genes in bacterial species genomes lacking CRISPR-CAS systems.

Table S5. Plasmid sequences present in bacterial species lacking CRISPR-CAS systems.

Species	Access number	N° Plasmids	Plasmid name
Aba. 11510	NZ_CP018861.2	1	Plasmid pAba11510a
Aur. ACS-120-V-Col10a	NC_015278.1	0	
Bbr. DZQ7	NZ_CP030117.1	0	
Bab. 21630	NZ_CP023235.1	0	
Cab. 1H	NZ_LN554883.1	0	
Cfr. R17	NZ_CP035276.1	1	Plasmid pCFR17_1
Cbo. Mfbjulcb6	CP027778.1	0	
Cps. Cap1R	NZ_CP036258.1	0	
Eic. MS-17-156	NZ_CP028813.1	2	Plasmid pEI-MS-17-156-1, Plasmid pEI-MS-17-156-2
*Eco.*PPECC42	NZ_CM003707.1	0	
Gni. OTC-16	NZ_CP033081.1	2	Plasmid unnamed1, Plasmid unnamed2
Hin. 5P54H1	NZ_CP020009.1	0	
Hpy. F209	NZ_AP017332.1	0	
Kpn. 121	NZ_CP031849.1	4	Plasmid pKP121-1-mcr, Plasmid pKP121-2, Plasmid pKP121-3, Plasmid pKP121-4
Lam. GRL 1112	NC_014724.1	2	Plasmid plasmid1, Plasmid plasmid2
*Lre.*ATG-F4	NZ_CP035790.1	0	
Lgr. NCTC 10812	NZ_LR134483.1	0	
Mlu. SB1254	NZ_CP026366.1	0	
Mab. FLAC013	NZ_CP014955.1	0	
Mle. TN	NC_002677.1	0	
Ppn. MCB032	NZ_CP015371.1	3	Plasmid unnamed 1, Plasmid unnamed 2, Plasmid unnamed 3
Pac. 6609	NC_017535.1	0	
Pae. Y71	NZ_CP030911.1	0	
Ppu. NX-1	NZ_CP030750.1	0	
Rty. str. TH1527	NC_017066.1	0	
Sen. SA20100345	NZ_CP022504.1	0	
Sha. SGAir0252	NZ_CP025031.1	1	Plasmid pSGAir0252B
Spn. NT_110_58	NZ_CP007593.1	0	
Spy. D471	NZ_CP011415.1	0	
Vch. C5	NZ_CP013301.1	0	

Table S6. Plasmid sequences present in bacterial species with confirmed CRISPR-CAS systems.

Species	Access number	N° Plasmids	Plasmid name
Ab. A388	NZ_CP024418.1	1	Plasmid pA388
Af. DSM 20731	NC_013740.1	0	
Bc. KSM-K16	NC_006582.1	0	
Bco. HM-08	NZ_CP010525.1	0	
Cj. NS4-5-1	NZ_CP007192.1	0	
Ec. K-12	NZ_LN832404.1	0	
Ecl. CZ-1	NZ_CP035738.1	0	
Ef. SRCM103470	NZ_CP035222.1	2	Plasmid unnamed1, Plasmid unnamed2
Efa. OG1RF	NC_017316.1	0	
Hi. NCTC11873	NZ_LR134490.1	0	
Hm. NCTC12198	NC_013949.1	0	
Kp. AATZP	NZ_CP014755.1	3	Plasmid pKPN-041, Plasmid pKPN-04f, Plasmid pNDM-1fa
La. YT1	NZ_CP025200.1	0	
Li. Clip11262	NC_003212.1	1	Plasmid pLI100
Lm. Lm3163	NZ_CP013722.1	0	
Mc. BBH18	NC_014147.1	0	
Mp. 1049	NZ_CP033058.2	0	
Na. NCTC12227	NZ_LR134516.1	0	
Nm. NCTC10025	NZ_LR134525.1	0	
Pa. UCBPP-PA14	NC_008463.1	0	
Pm. FDAARGOS_67	NZ_CP026051.1	0	
Sd. CFSAN010956	NZ_CP026827.1	1	Plasmid unnamed
Se. FDAARGOS_153	NZ_CP014119.1	4	Plasmid unnamed1, Plasmid unnamed2, Plasmid unnamed3, Plasmid unnamed4
Sen. SA19980677	NZ_CP007285.2	0	
Sm. N4-5	NZ_CP031316.1	1	Plasmid pSmN45
Sp. MGAS23530	CP013839.1	0	
Vc. FORC_076	NZ_CP026531.1	0	
Vp. FORC_022	NZ_CP013249.1	1	Plasmid unnamed
Vv. YJ016	NC_005140.1	1	Plasmid pYJ016
Yp. FDAARGOS_602	NZ_CP033696.1	2	Plasmid unnamed1, Plasmid unnamed2

Table S7. Plasmid sequences present in archaeal species with confirmed CRISPR-CAS systems.

Species	Access number	N° Plasmids	Plasmid name
Af. DSM_4304	NC_000917.1	0	
Ap. K1	NC_000854.2	0	
Cd. PM4	NZ_LT719092.1	0	
Fa. Fer1	NC_021592.1	0	
Fp. DSM_10642	NC_013849.1	0	
Ga. SBH6	NZ_CP009552.1	0	
Hb. DSM 5456	NC_008818.1	0	
Hli. tADL	NZ_CP024845.1	0	
Ia. DSM 17230	NC_014471.1	0	
Ih. KIN4/I	NC_009776.1	0	
Ma. MRE50	NC_009464.1	0	
Mac. C2A	NC_003552.1	0	
Mb. MS2	NC_018227.2	0	
Mf. AG86	NC_013156.1	1	Plasmid pMEFER01
Mh. JF-1	NC_007796.1	0	
Mi. Kol5	NC_015562.1	0	
Mp. E1-9c	NC_011832.1	0	
Ms. ATCC35061	NC_009515.1	0	
Mst. DSM_3091	NC_007681.1	0	
Nca. SCU2	NZ_LT981265.1	0	
Ne. SR1	NZ_CP007174.1	0	
Pd. Su06	NZ_CP013011.1	0	
Pf. 1A	NC_015931.1	0	
Pt. DSM9790	NC_005877.1	0	
Sa. HS-1	NZ_AP018553.1	0	
Si. L.S.2.15	NC_012589.1	0	
Ss. SULA	NZ_CP011057.1	0	
Tt. Kra1	NC_016070.1	0	
Tv. GSS1	NC_002689.2	0	
Vm. 768-28	NC_015151.1	0	
